# Physiological dynamics of chemosynthetic symbionts in hydrothermal vent snails

**DOI:** 10.1038/s41396-020-0707-2

**Published:** 2020-07-02

**Authors:** Corinna Breusing, Jessica Mitchell, Jennifer Delaney, Sean P. Sylva, Jeffrey S. Seewald, Peter R. Girguis, Roxanne A. Beinart

**Affiliations:** 1 University of Rhode Island, Graduate School of Oceanography, Narragansett, RI USA; 2 Harvard University, Department of Organismic and Evolutionary Biology, Cambridge, MA USA; 3 Woods Hole Oceanographic Institution, Department of Marine Chemistry and Geochemistry, Woods Hole, MA USA

**Keywords:** Metabolism, Symbiosis

## Abstract

Symbioses between invertebrate animals and chemosynthetic bacteria form the basis of hydrothermal vent ecosystems worldwide. In the Lau Basin, deep-sea vent snails of the genus *Alviniconcha* associate with either *Gammaproteobacteria* (*A. kojimai*, *A. strummeri*) or *Campylobacteria* (*A. boucheti*) that use sulfide and/or hydrogen as energy sources. While the *A. boucheti* host–symbiont combination (holobiont) dominates at vents with higher concentrations of sulfide and hydrogen, the *A. kojimai* and *A. strummeri* holobionts are more abundant at sites with lower concentrations of these reductants. We posit that adaptive differences in symbiont physiology and gene regulation might influence the observed niche partitioning between host taxa. To test this hypothesis, we used high-pressure respirometers to measure symbiont metabolic rates and examine changes in gene expression among holobionts exposed to in situ concentrations of hydrogen (H_2_: ~25 µM) or hydrogen sulfide (H_2_S: ~120 µM). The *campylobacterial* symbiont exhibited the lowest rate of H_2_S oxidation but the highest rate of H_2_ oxidation, with fewer transcriptional changes and less carbon fixation relative to the *gammaproteobacterial* symbionts under each experimental condition. These data reveal potential physiological adaptations among symbiont types, which may account for the observed net differences in metabolic activity and contribute to the observed niche segregation among holobionts.

## Introduction

Symbiotic microbes are increasingly recognized as important drivers of animal development, physiology and speciation [1, 2]. How symbionts influence the biology of their host is particularly evident in nutritional symbiotic relationships, which often broaden the ecological niche of the animal partner by providing access to novel resources, thereby fostering both ecological and evolutionary diversification [3–7]. Symbiont-mediated niche expansion, habitat partitioning and local adaptation have been well described in insect-microbe and coral-algae symbioses [3–5], but these processes are less understood in other symbiotic systems.

Mutualistic symbioses between chemosynthetic bacteria and invertebrate animals at deep-sea hydrothermal vents offer intriguing opportunities to study the effects of symbiont physiology on host adaptation and niche segregation in the ocean, given the intricate links between geochemical environment, symbiont metabolism and host survival. In these associations, the symbiotic bacteria oxidize reduced chemicals present in the hydrothermal fluids—e.g., sulfide (H_2_S), methane (CH_4_) and/or hydrogen (H_2_)—to gain energy for the fixation of inorganic carbon into organic matter, which provides the bulk of nutrition for the animal host (chemosynthesis; [8]). Chemosynthetic symbioses have evolved many times: invertebrate hosts come from divergent taxonomic groups within three invertebrate phyla, while their symbionts have evolved from several distinct lineages of two bacterial phyla [8]. Host and symbiont taxa typically show a strong selectivity towards each other, as each host species associates with only one to a few distinct bacterial symbiont lineages [8]. Thus, given that symbiont phylogenetic diversity can correspond to functional diversity, ecological differences among host taxa may be tied to the specific traits of their symbiont(s).

Diversity in chemosynthetic traits among symbionts may be especially important to habitat segregation in these systems. Chemosynthetic host–symbiont associations (holobionts) are often characterized by heterogeneous distributions at both local and regional scales, with discrete patches or zones of animal taxa occurring within a single-vent field or entire fields being dominated by one or a few taxa [9–11]. Since the composition and concentration of fluid compounds for chemosynthetic processes can vary substantially over temporal and spatial scales [12], the distribution of many holobionts seems to be linked to taxon-specific associations with particular habitat characteristics. Thus, local and regional community composition, and ultimately the overall biological diversity, may be strongly influenced by the partitioning of holobionts into distinct physico-chemical niches. Yet, to date, the mechanistic links between fluid geochemistry and holobiont community composition are poorly known.

Snails of the genus *Alviniconcha* provide a unique opportunity to understand symbiont-meditated habitat partitioning at hydrothermal vents [13, 14]. In the Eastern Lau Spreading Center (ELSC), Tonga, three species of *Alviniconcha* (*A. boucheti*, *A. kojimai*, *A. strummeri*) co-occur at several vent localities [13, 15], where they establish endosymbioses with three different lineages of chemoautotrophic *Gammaproteobacteria* or *Campylobacteria* (formerly *Epsilonproteobacteria*; [16]). The bacterial symbionts are assumed to be environmentally acquired given that phylogenetic studies suggest an absence of host–symbiont co-evolution [13, 17]. Nevertheless, host species and symbiont 16S ribosomal RNA (rRNA) phylotypes appear to be highly selective towards each other: *A. boucheti* typically harbors a *campylobacterial* symbiont phylotype (called ε), whereas *A. kojimai* and *A. strummeri* associate with two distinct lineages of *gammaproteobacterial* symbiont phylotypes (called γ-1 and γ-Lau) [13].

Beinart et al. [13] suggested that the distribution of different host–symbiont combinations is determined by local and regional variations in vent geochemistry. While *A. boucheti* holobionts are predominantly found at northern sites characterized by high-fluid concentrations of H_2_S and H_2_, the *A. kojimai* and *A. strummeri* holobionts are more frequently observed at southern sites where concentrations of these reductants are lower (Supplementary Table S1). The authors hypothesized that differences in symbiont metabolic potential might drive the differential abundance of symbiont and host genotypes. Subsequent genome comparisons between the three symbiont lineages showed that these symbionts are broadly similar in terms of gene content related to chemolithoautotrophy, although differences exist in the specific encoded enzyme types and metabolic pathways [18]. The large overlap in overall metabolic potential but discrepancy in realized pathways implies that differences in enzyme kinetics and gene regulation might underlie the observed habitat partitioning between snail holobionts [18]. To address this question, we supplied the ELSC *Alviniconcha* holobionts with defined concentrations of dissolved sulfide and hydrogen in high-pressure shipboard experiments and measured changes in metabolic rates and gene expression. Exposing the symbionts to the same environmental conditions allowed us to compare the holobionts’ transcriptional responses and chemosynthetic activities, thereby providing insights into potential adaptations that are likely important to their realized distribution across a range of geochemical habitats.

## Materials and methods

### Sample collection and experimental setup

*Alviniconcha* were collected with the ROV *ROPOS* during the R/V *Falkor* expedition FK160409 in 2016 (Table 1). Snails were recovered in insulated containers and briefly kept in filtered, 4 °C seawater. *Alviniconcha* species were morphologically identified and only those responsive to touch were used for experiments. All experiments were conducted under in situ pressure (~20.68 MPa) at a temperature of 15–17 °C in three custom-built, titanium flow-through aquaria that are part of a high-pressure respirometry system (HPRS; Supplementary Methods).

**Table 1:** Summary of the six experiments presented here, including the reductant supplied, geographic coordinates and depths from which the snail species were collected, as well as the number (*N*) of control and treatment animals used in each experiment.

Exp.	Reductant	Vent field	Latitude	Longitude	Depth (m)	Species	Symbiont	Control (*N*)	Treatment (*N*)
1	H_2_S	ABE	–20.7630	–176.1913	2155	*A. boucheti* *A. kojimai*	ε γ-1	2 2	4 4
2	H_2_S	Tu’i Malila	–21.9891	–176.5682	1884	*A. strummeri* *A. kojimai*	γ-1 γ-1	3 3	4 4
3	H_2_	Tu’i Malila	–21.9891	–176.5682	1888	*A. strummeri* *A. kojimai*	γ-1 γ-1	3 3	4 5
4	H_2_	ABE	–20.7617	–176.1917	2130	*A. boucheti* *A. kojimai*	ε γ-1	2 2	4 5
5	H_2_	Tahi Moana	–20.6838	–176.1834	2214	*A. strummeri* *A. boucheti*	γ-1 ε	3 3	3 3
6^a^	None	Tow Cam	–20.3166	–176.1361	2703	*A. boucheti* *A. kojimai*	ε γ-1	0 0	3 3

^a^As we were unable to include an acclimation control, transcriptomic sequencing was not performed for individuals in this experiment.

### Hydrogen and hydrogen-sulfide experiments

A total of six experiments were conducted, two with hydrogen sulfide, three with hydrogen, and one with no reductant (Table 1 and Supplementary Fig. S1). Each experiment compared two *Alviniconcha* species, with 3–5 individuals of each species being placed separately into two of the three aquaria, and 2–3 individuals of each species being placed together into the third aquarium for use as the “acclimation control”. Each experiment started with a 24 h acclimation period in the HPRS, where snails were kept in oxygenated seawater (~250–295 µM O_2_) without exposure to reductant or nitrate. The control aquarium was depressurized at the conclusion of the acclimation period, and snails of both species were quickly weighed, dissected, and sampled for measurement of carbon stable isotopic composition and transcriptomic sequencing. Whenever possible, this third aquarium was re-pressurized, and utilized as an empty chemistry control to quantify abiotic losses of reductant and oxygen in the HPRS system during the experiments. Alternatively, a separate control experiment was conducted (“estimated control”; Supplementary Methods; Supplementary Fig. S2). Following the acclimation period, the aquaria (except for Experiment 6) were exposed to a 24 h treatment with a chemical reductant (~25 µM H_2_ or ~120 µM H_2_S) and nitrate (~40 µM), after which the snails were processed for further analyses as described above. The H_2_S concentration was chosen based on previous experiments with *Alviniconcha* [19] and the H_2_ concentration was the maximum concentration that could be achieved utilizing a Parker Balston H2-90 H_2_ generator.

### Oxidation, respiration, and carbon incorporation rates

Hydrogen, sulfide, and oxygen concentrations were measured in the input and effluent from each aquarium as described in the Supplementary Methods. Mass-specific H_2_ and H_2_S oxidation and O_2_ respiration rates were calculated as in ref. [19] by comparing the steady-state concentrations of H_2_, H_2_S, and O_2_ between effluents from treatment and empty control aquaria (Supplementary Tables S2 and S3; and Supplementary Figs. S3 and S4). In the experiments without empty control aquarium, the control concentrations were estimated by correcting the input water concentration by the mean proportion of abiotic reductant loss.

After the acclimation period, input seawater was amended with ^13^C labeled sodium bicarbonate (Na^13^CO_3_; 99.9% atom percent; Cambridge Isotopes Laboratories, Inc.) to achieve a final ^13^C atom percent (A%) of 2–4%. Sample processing and calculation of ^13^C incorporation rates (^13^C_inc_) was performed as in ref. [19], except that instead of normalizing to the A% of experimental foot tissue, the average gill A% for acclimation animals of the same species was used, which provides a better approximation of the initial natural stable isotopic composition.

### Sequencing and bioinformatic analyses

At the end of the acclimation and experimental periods, gill tissue pieces were excised from each animal for host mitochondrial *COI* genotyping and transcriptomic sequencing as detailed in the Supplementary Methods. *COI* gene sequences have been deposited in GenBank under accession numbers MN551348-MN551413, while raw RNAseq reads have been uploaded to the Sequence Read Archive under BioProject number PRJNA526236. After initial quality checks, raw reads were trimmed, filtered for rRNA, host and other contaminating sequences, and mapped against the *Alviniconcha* symbiont genomes [18] for symbiont read quantification (Supplementary Methods). Since read support for minority symbionts was low (usually <1,000,000 reads), we focused our analyses on the most abundant endosymbiont in each host species to ensure accurate transcript estimation and statistical robustness (Table 1 and Supplementary Table S4). For each sample, transcripts of the dominant symbiont were mapped against the RASTtk annotated gene set of the symbiont’s draft genome with BBMAP (https://sourceforge.net/projects/bbmap/) and then quantified with SALMON [20]. General transcription profiles for each sample were obtained by calculating Trimmed Mean of M normalized Transcripts Per Million (TPM) values [21, 22] for individual transcripts as well as broader gene categories that we defined based on the RASTtk annotations and literature searches. Differential gene expression between control and treatment samples for each species and experimental setup was identified with *DESeq2* in R [23, 24], accounting for batch effects and pseudo-replication and adjusting p-values for type I error (Supplementary Methods).

## Results

### Oxidation and respiration rates

**Fig. 1:**
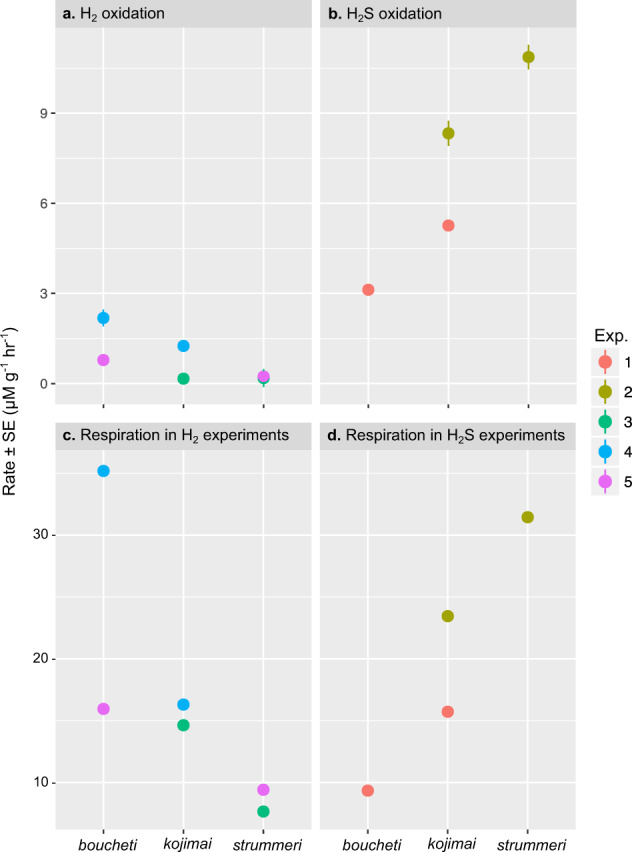
Oxidation and respiration rates for *Alviniconcha* species. Mean mass-specific oxidation rates for (**a**) H_2_ and (**b**) H_2_S, as well as the mass-specific O_2_ respiration rates for *Alviniconcha* species while oxidizing (**c**) H_2_ or (**d**) H_2_S. All mass-specific rates are expressed as per gram of wet gill tissue and their standard error are given.

All three *Alviniconcha* holobionts demonstrated the ability to oxidize H_2_S and H_2_ (Fig. 1a, b and Supplementary Table S2). The rates of H_2_S and H_2_ oxidation showed opposing patterns for two of the species, with *A. boucheti* having the highest mass-specific rate of H_2_ oxidation, but lowest rate of H_2_S oxidation, and vice versa for *A. strummeri* (Fig. 1a, b). *A. kojimai* had H_2_ and H_2_S oxidation rates in between the two other species (Fig. 1a, b). Oxygen respiration rates followed the same pattern (Fig. 1c, d and Supplementary Table S3). These findings were unrelated to animal size as Spearman Rank correlations of gill weight with H_2_S/H_2_ oxidation rate and O_2_ respiration rate were insignificant.

### Inorganic carbon incorporation rates

**Fig. 2:**
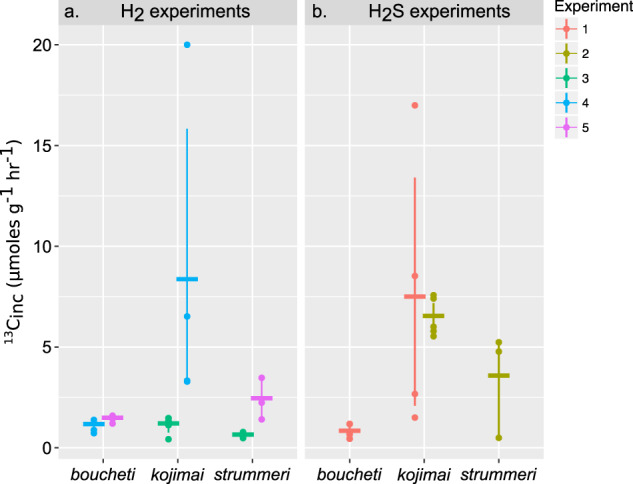
Carbon incorporation rates for *Alviniconcha*. Mass-specific carbon incorporation rates (^13^C_inc_) for *Alviniconcha* individuals when oxidizing (**a**) H_2_ or (**b**) H_2_S. Horizontal bars show the mean for each experiment and error bars show the 95% confidence intervals. All mass-specific rates are expressed as per gram of wet gill tissue.

All individuals from the experiments with reductant showed enrichment in ^13^C to levels above the natural abundance threshold (Supplementary Fig. S5; and Supplementary Tables S5 and S6). However, ^13^C_inc_ did not directly follow H_2_ or H_2_S oxidation rates (Fig. 2). ^13^C_inc_ for *A. boucheti* was generally low, but similar among the H_2_ and H_2_S treatments (Fig. 2). *A. kojimai* demonstrated higher ^13^C_inc_ than *A. strummeri* under both conditions, though many *A. kojimai* and *A. strummeri* individuals exhibited comparable ^13^C_inc_ when oxidizing H_2_ or H_2_S (Fig. 2). For reasons that are unclear, individuals of both *A. kojimai* and *A. strummeri* exhibited markedly low rates of H_2_ oxidation, O_2_ respiration, and carbon incorporation during Experiment 3. The observed patterns were not linked to animal size with the exception of Experiment 4 where some larger *A. kojimai* individuals exhibited unusually high-carbon incorporation rates (*S* = 19.616, *p*-value = 0.02652; Supplementary Table S6).

In Experiment 6, where *A. boucheti* and *A. kojimai* were exposed to ^13^C label in the absence of any exogenous reductant, A% values were above the natural abundance threshold for *A. kojimai* across experiments, indicative of inorganic carbon fixation (Supplementary Fig. S5 and Supplementary Table S6). Owing to the lack of an acclimation control, we are unable to calculate precise mass-specific ^13^C incorporation rates in this experiment. However, using the mean acclimation A% for each species as an approximation of our initial carbon isotope ratio, we calculate a mean estimated rate of 2.22 µmoles g^−1^ h^−1^ for *A. kojimai* and no incorporation for *A. boucheti*.

### Symbiont transcription profiles

High-throughput RNA sequencing on the Illumina™ Nova- and NextSeq instruments resulted in an average of 43,960,734 paired-end reads per sample, 5,382,133 (12.24%) of which remained for analysis. The dominant symbiont phylotype represented at least 75% of all symbiont reads in each snail individual: γ-1 in *A. kojimai* and *A. strummeri*, and ε in *A. boucheti* (Table 1 and Supplementary Table S4). The remaining symbiont reads were comprised of either γ-1 (in *A. boucheti*) or ε (in *A. kojimai* and *A. strummeri*), while the γ-Lau symbiont was lowly abundant (<5%) in all samples (Supplementary Table S4).

**Fig. 3:**
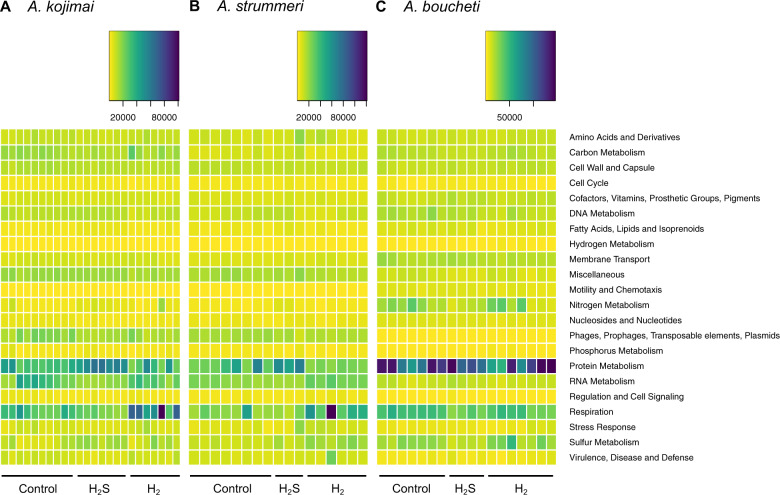
Transcriptome profiles for *Alviniconcha* species. Transcriptome profiles for the (**a**) *A. kojimai*, (**b**) *A. strummeri* and (**c**) *A. boucheti* symbiont. Hypothetical genes are excluded from this plot for better visibility of gene expression status in other categories (see also Supplementary Fig. S6). Plotted values are TMM normalized TPMs.

Independent of experimental treatment, the symbionts of all *Alviniconcha* species exhibited the highest expression levels for hypothetical genes, followed by genes involved in protein metabolism and respiration (Fig. 3 and Supplementary Fig. S6). A moderate to high expression was also observed for RNA metabolism genes in the γ-1 symbiont of *A. kojimai* and *A. strummeri*, and for sulfur and nitrogen metabolism genes in the ε symbiont of *A. boucheti*. Key genes involved in sulfur oxidation and nitrogen metabolism constituted 2.02–3.32% and 3.80–6.01% of all transcripts in the ε symbiont, but only 1.32–2.46% and 0.45–1.71% in the γ-1 symbiont, respectively (Supplementary Table S7). Transcripts for hydrogen oxidation genes were lowly abundant in all symbiont types (0.00–0.04%; Supplementary Table S7). Transcripts for carbon fixation genes comprised 1.44–1.68% in the ε symbiont, which uses the reverse tricarboxylic acid (rTCA) cycle for carbon assimilation [18], and 0.12–0.33% in the γ-1 symbiont, which uses the Calvin-Benson-Bassham (CBB) cycle [18] (Supplementary Table S7).

### Symbiont differential gene expression: sulfide treatment

**Fig. 4:**
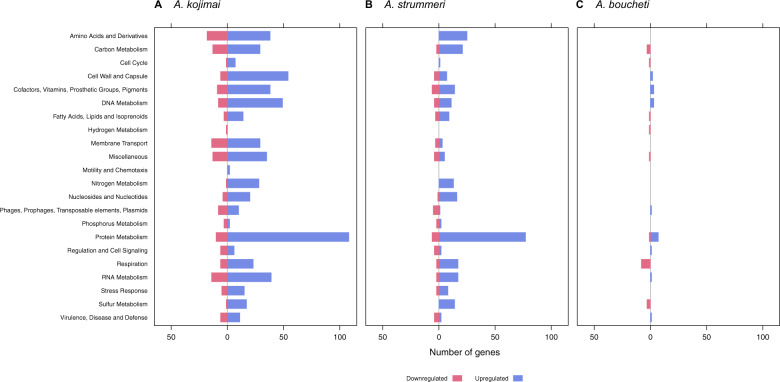
Differential gene expression in the sulfide treatment. Differential gene expression in the sulfide treated symbionts of (**a**) *A. kojimai*, (**b**) *A. strummeri*, and (**c**) *A. boucheti* based on an adjusted *p*-value of 0.05. Hypothetical genes are excluded from this plot for better visibility of gene expression changes in other categories (see also Supplementary Fig. S7). Comparisons are relative to the acclimation control samples.

**Fig. 5:**
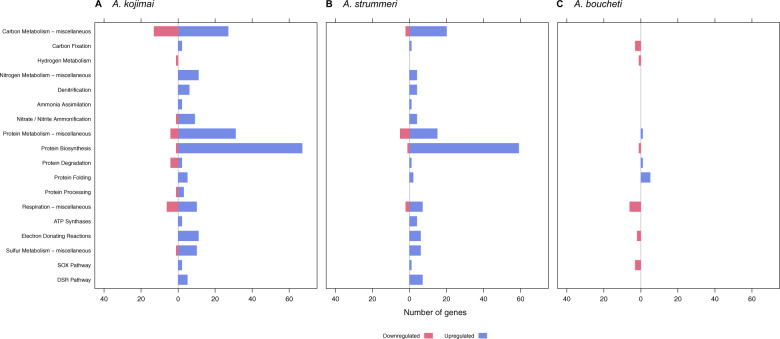
Differential gene expression for selected functional categories in the sulfide treatment. Differential gene expression for metabolic subcategories in the sulfide treated symbionts of (**a**) *A. kojimai*, (**b**) *A. strummeri*, and (**c**) *A. boucheti* based on an adjusted *p*-value of 0.05. Comparisons are relative to the acclimation control samples.

In the *A. kojimai*–γ-1 holobiont, 1776 genes were differentially expressed (Supplementary Tables S8 and S9). One-thousand fifty-two of these genes were hypothetical proteins, most of which were downregulated (Supplementary Fig. S7a). Differentially expressed (DE) genes in other categories, especially protein (118), cell wall (60), DNA (57), amino acid (56), RNA (53), and cofactor (47) metabolism, showed opposing patterns (Fig. 4a). Many of these genes were functionally related to biosynthetic processes, such as ribosome assembly, transcription and biogenesis of extra- and intracellular compounds (Fig. 5a and Supplementary Table S9). Likewise, DE genes involved in carbon (42) and nitrogen (29) metabolism (e.g., CO_2_ uptake, gluconeogenesis, denitrification and nitrate/nitrite ammonification) showed an increase in expression (Figs. 4a and 5a). Seventeen genes related to sulfur metabolism were upregulated, including *soxZ*, *soxY*, genes of the sulfite reduction-associated complex DsrMKJOP, rhodanese-related sulfurtransferases, sulfide:quinone oxidoreductase (SQR) type I, adenylylsulfate reductase, sulfate adenylyltransferase, and thioredoxin-disulfide reductase (Fig. 5a and Supplementary Table S9). Expression of hydrogen metabolism genes did not change, except for a quad-[4Fe-4S] ferredoxin of the HycB/HydN/HyfA family, which was downregulated.

Gene expression patterns in the *A. strummeri* –γ-1 holobiont mirrored those in the *A. kojimai* –γ-1 holobiont, although only 422 genes were differentially expressed (Supplementary Tables S8 and S9). Most DE genes encoded hypothetical proteins (103), which were largely downregulated (Supplementary Fig. S7b). DE genes related to biosynthetic pathways, especially protein (83), amino acid (25), cofactor (20), and RNA (19) metabolism, as well as DE genes involved in carbon (22) and nitrogen (13) metabolism were predominantly upregulated (Fig. 4b and Supplementary Table S9). These genes included various enzymes related to inorganic carbon assimilation, denitrification, and nitrate/nitrite ammonification (Fig. 5b and Supplementary Table S9). Fourteen sulfur metabolism genes showed an increase in expression, including *soxA*, SQR type VI, sulfate adenylyltransferase, genes of the sulfite reduction-associated complex DsrMKJOP and adenylylsulfate reductase (Fig. 5b and Supplementary Table S9). Hydrogen metabolism genes showed no change in expression.

In the *A. boucheti*–ε holobiont, 66 genes were differentially expressed (Supplementary Tables S8 and S9). These DE genes encoded mostly hypothetical proteins (28), which were upregulated (Supplementary Fig. S7c). Likewise, DE genes associated with protein metabolism, in particular heat shock proteins and chaperones, were upregulated, whereas DE genes involved in cellular respiration were downregulated (Figs. 4c and 5c; and Supplementary Table S9). Three genes related to sulfur oxidation (*soxD*, *soxY*, sulfur oxidation molybdopterin C protein), one gene related to hydrogen oxidation (quinone-reactive Ni/Fe-hydrogenase large chain), and three genes of the rTCA cycle (2-oxoglutarate/2-oxoacid ferredoxin oxidoreductase subunits β, γ, δ) were downregulated (Fig. 5c and Supplementary Table S9). No changes in expression were seen for nitrogen metabolism genes.

### Symbiont differential gene expression: hydrogen treatment

**Fig. 6:**
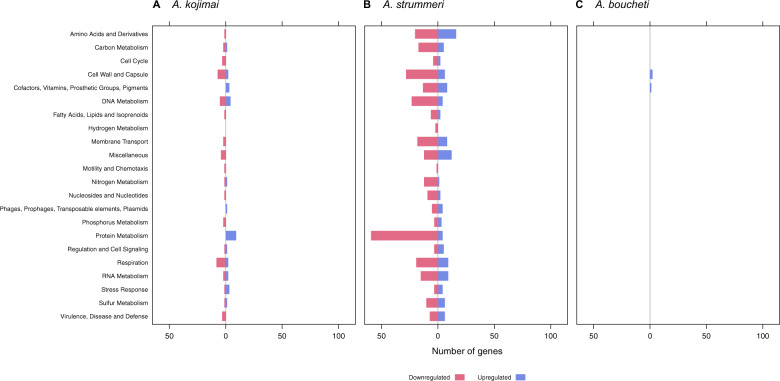
Differential gene expression in the hydrogen treatment. Differential gene expression in the hydrogen treated symbionts of (**a**) *A. kojimai*, (**b**) *A. strummeri*, and (**c**) *A. boucheti* based on an adjusted *p*-value of 0.05. Hypothetical genes are excluded from this plot for better visibility of gene expression changes in other categories (see also Supplementary Fig. S8). Comparisons are relative to the acclimation control samples.

**Fig. 7:**
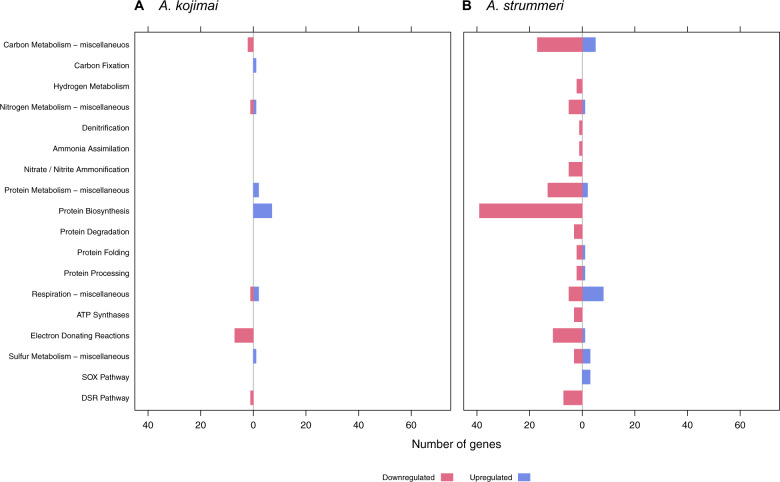
Differential gene expression for selected functional categories in the hydrogen treatment. Differential gene expression for metabolic subcategories in the hydrogen treated symbionts of (**a**) *A. kojimai* and (**b**) *A. strummeri* based on an adjusted p-value of 0.05. No significant changes in expression were seen in the *A. boucheti* symbiont for these categories. Comparisons are relative to the acclimation control samples.

In the hydrogen treated *A. kojimai*–γ-1 holobiont 435 genes were differentially expressed (Supplementary Tables S8 and S9). Again, the majority of these genes comprised hypothetical proteins (359), although these were predominantly upregulated (Supplementary Fig. S8a). DE genes involved in respiration (10) and cell wall functions (9) showed mostly a decrease in expression, while genes involved in protein metabolism (9), especially biosynthesis of the ribosomal complex, showed an increase in expression (Figs. 6a and 7a; and Supplementary Table S9). Three genes related to carbon metabolism and two genes related to nitrogen metabolism were differentially expressed. Of these, ribulose bisphosphate carboxylase and the heme d1 biosynthesis proteins NirD/NirL were upregulated (Fig. 7a and Supplementary Table S9). In the sulfur oxidation pathway, the gene encoding the sulfite reduction-associated complex DsrMKJOP iron-sulfur protein DsrO was downregulated, while anaerobic dimethyl sulfoxide reductase chain A was upregulated. Expression of hydrogen oxidation genes did not change.

In the *A. strummeri*–γ-1 holobiont, 1113 genes were differentially expressed (Supplementary Tables S8 and S9). Most of these genes encoded hypothetical proteins, which were upregulated (615; Supplementary Fig. S8b). DE genes in other categories, especially protein metabolism (59), cell wall and capsule (28), and DNA metabolism (23), showed mostly a decrease in expression (Fig. 6b). Similarly, DE genes involved in carbon, nitrogen and hydrogen metabolism (e.g., HyaF, HyaB) and the reverse dissimilatory sulfite reductase (DSR) pathway were downregulated, whereas genes of the SOX pathway were upregulated (Fig. 7b and Supplementary Table S9).

In the hydrogen treated *A. boucheti*–ε holobiont, only nine genes were differentially expressed (Supplementary Tables S8 and S9), especially hypothetical proteins (6), which were upregulated (Supplementary Fig. S8c). Additionally, one gene involved in cofactor biosynthesis and two genes involved in cell wall metabolism showed an increase in expression, but no significant differences were found in any other categories (Fig. 6c).

## Discussion

Animal–microbe symbioses at hydrothermal vents are opportune systems to study the links between environmental conditions, symbiont metabolism and host biogeography. Among sympatric *Alviniconcha* vent snail species of the ELSC, the traits of their chemosynthetic bacterial symbionts are hypothesized to influence their distribution and niche segregation [13, 14], but the underlying physiological and genetic mechanisms have not yet been examined in detail. Here, we performed high-pressure shipboard experiments to assess differences in symbiont metabolic rates and transcriptional regulation that might play a role in symbiont-mediated host niche partitioning of the co-occurring species *A. kojimai*, *A. strummeri*, and *A. boucheti*.

Our results indicated that the *campylobacterial* ε symbiont and the *gammaproteobacterial* γ-1 symbiont show contrasting metabolic and transcriptional responses to the offered concentrations of hydrogen and sulfide. Although any net metabolic measurements of the holobiont represent the activities of all symbiotic bacteria, the ε and γ-1 phylotypes comprised about 75–80% of the symbiont metatranscriptome in *A. boucheti* and *A. kojimai*/*A. strummeri*, respectively (Supplementary Table S4), and, thus, likely account for the physiological responses observed here. The presence of a single dominant phylotype in each snail host is consistent with a previous quantitative assessment of *Alviniconcha* symbiont communities [13], while intracellular minority symbiont populations have not yet been confirmed microscopically. Therefore, it is possible that the minority phylotypes (~20% γ-1 in *A. boucheti* and ~20% ε in *A. kojimai*/*A. strummeri*) detected here are actually free-living symbionts attached to gill surface or other contaminants. However, if these represent true minority symbiont populations, they have the potential to contribute to the physiological plasticity of these holobionts and might increase their adaptability to environmental fluctuations. Interestingly, the γ-Lau symbiont comprised only a small percentage of the symbiont community in our experimental *Alviniconcha*, despite having been previously seen to be abundant in *A. strummeri* from the Tu’i Malila vent field [13]. These findings may reflect intra-field patchiness in the frequency of associations between *A. strummeri* and the two *gammaproteobacterial* symbiont phylotypes.

Although we were unable to obtain geochemical measurements directly from the snail patches sampled here, reductant concentrations in end-member hydrothermal fluids and *Alviniconcha* beds were measured on previous expeditions (Supplementary Table S1). End-member concentrations do not represent the habitat conditions that these snails experience, but give a relative approximation of the chemical gradient that exists between vent sites. The *A. boucheti* holobiont dominates at hydrothermal vents with high sulfide and hydrogen concentrations, and the experimental concentrations used here were likely in the normal to lower range of what they typically experience [13, 25] (Supplementary Table S1). However, for the *A. kojimai* and *A. strummeri* holobionts, the experimental conditions likely represented values that are on the high end of what they normally encounter [13, 25] (Supplementary Table S1). Adaptations to these conditions may explain the distinct metabolic and transcriptional patterns we observed.

*A. boucheti*’s ε symbiont oxidized H_2_S at the lowest and H_2_ at the highest rates, but incorporated less carbon than the γ-1 symbiont under both experimental conditions. Interestingly, carbon incorporation rates were comparable in the *A. boucheti* symbiont when oxidizing either reductant, even though rates of sulfide oxidation were almost twice the rates of hydrogen oxidation. This greater efficiency of hydrogen oxidation is consistent with both growth experiments and a recent theoretical model of chemosynthetic efficiency in *Sulfurimonas denitrificans*, a close relative of the ε symbiont, which suggests that productivity via aerobic hydrogen oxidation is at least three times higher than via aerobic sulfur oxidation [26, 27].

The *A. boucheti* symbiont showed no significant changes in expression for genes related to sulfur and hydrogen oxidation, carbon fixation, and nitrogen metabolism under the experimental hydrogen treatment, and started to downregulate a few of these genes under the experimental sulfide treatment. It is possible that this ε symbiont constitutively expresses some key genes involved in chemoautotrophy, which supports recent views of transcriptional regulation in chemosynthetic *Sulfurimonas* [28]. For example, a core enzyme involved in sulfide oxidation is sulfide:quinone reductase, which is present as SQR type IV and VI in the genomes and the studied transcriptomes of the *A. boucheti* symbiont. In non-symbiotic *Sulfurimonas* these SQR types have optimal work H_2_S concentrations of >2 mM and >4 mM, respectively [29]. SQR type IV is assumed to be constitutively expressed, while SQR type VI is only activated at high sulfide concentrations. Although it is unknown how these SQRs are regulated in the *A. boucheti* symbiont, it is possible that they function in a similar way as those of free-living sulfide-oxidizing *Sulfurimonas*. This could explain why we did not see significant expression changes in these SQR types and why the *A. boucheti* symbiont exhibited relatively low rates of H_2_S oxidation. Notably, this symbiont increased expression of heat shock proteins and chaperones, implying that it was mitigating some stressor at the experimental sulfide conditions. This may indicate that the provided amount of sulfide was low relative to in situ concentrations, or that the ε symbiont prefers access to both hydrogen and sulfide at the same time. For example, free-living *Campylobacteria* have transcriptional patterns that suggest that they utilize many reductants simultaneously instead of preferentially [28], while growth rates are highest when both hydrogen and sulfur are accessible [26]. Consequently, the *A. boucheti*–ε holobiont may be adapted to the presence of high concentrations of both sulfide and hydrogen in its habitat and may have arrested its metabolism under the experimental conditions used here. These assumptions are supported by comparisons with in situ gene expression patterns of the ε symbiont [14]: natural transcript abundances for sulfur, nitrogen and carbon metabolism genes showed closer resemblance to transcript abundances in the experimental control group than to those in the treatment groups. The ε symbiont assimilates inorganic carbon via the rTCA cycle, which is more energy efficient than the CBB cycle used by the γ-1 symbiont [30]. Metabolic arrest could explain why carbon was incorporated less effectively by the ε symbiont despite the higher efficiency of its carbon fixation metabolism.

We observed no change in expression for hydrogen oxidation genes in the *A. boucheti* symbiont under the experimental H_2_ conditions. These findings differ from a recent study by Miyazaki et al. [31], which showed that the *campylobacterial* symbiont of *A. marisindica* strictly regulates the expression of hydrogenases depending on the environmental hydrogen concentrations. Compared to Miyazaki et al. [31], who provided 100 µM H_2_ in their experiments, we only provided 25 µM H_2_. Thus, it is possible that the experimental hydrogen concentration supplied in our study was insufficient to induce upregulation of hydrogenases in the *A. boucheti* symbiont. An alternative, though not mutually exclusive, explanation could be that there are taxon-specific differences in hydrogenase regulation, since the *A. marisindica* symbiont belongs to the genus *Sulfurovum* [31], while the *A. boucheti* symbiont is related to the genus *Sulfurimonas* [13]. Free-living *Sulfurovum* are known to elevate hydrogenase activity under high H_2_ concentrations [32], while varied responses have been observed in *Sulfurimonas* [29]. For example, of the two free-living *Sulfurimonas* species occurring at hydrothermal vents, only one was shown to consume hydrogen under experimental conditions [29].

Very different reactions were observed in the γ-1 symbiont of *A. kojimai* and *A. strummeri*, which showed higher rates of sulfide oxidation and carbon fixation, but lower rates of hydrogen oxidation than the ε symbiont. When exposed to dissolved sulfide, the γ-1 symbiont upregulated several genes involved in sulfur oxidation, nitrate respiration and assimilation, carbon fixation, and biosynthetic processes and showed transcript abundances that were similar to in situ conditions [14]. In contrast to the *A. boucheti* symbiont, this endosymbiont possesses SQR types I and VI. While little is known about the enzyme kinetics of SQR type I, our data indicate that it is active at lower H_2_S levels, thereby allowing efficient coupling of sulfide oxidation and carbon incorporation under the experimental conditions. SQR type I was significantly upregulated in the γ-1 symbiont of *A. kojimai*, which showed the highest rates of carbon fixation in the sulfide treatment. Interestingly, in *A. strummeri* the γ-1 symbiont increased expression of SQR type VI, which is a high-sulfide adapted enzyme [29]. The contrasting response of this symbiont when associated with different host species may suggest adaptive differences to their specific habitats, which might be related to potential strain-level differences in the populations of γ-1. Among the three *Alviniconcha* species, *A. strummeri* is primarily found at sites with the lowest concentrations of sulfide [13]. The upregulation of a high-sulfide adapted enzyme suggests that the experimental sulfide concentrations were at the upper end of what *A. strummeri* naturally experiences and that their SQR type VI may have evolved to function at this substrate level threshold.

When exposed to dissolved hydrogen, the γ-1 symbiont of *A. kojimai* and *A. strummeri* oxidized hydrogen at a lower rate than sulfide, while carbon incorporation rates were similar between treatments. This may indicate that coupling of H_2_ oxidation and biomass production was more efficient in this symbiont than in the ε symbiont at the provided hydrogen concentrations, possibly due to differences in energetic efficiencies between different carbon fixation pathways [30, 33]. However, there is also plausible evidence that chemosynthetic energy might have been acquired from an additional, endogenous source. For example, although all experimental snails were acclimated without reductant for 24 h prior to experimentation, some of the “net” carbon incorporation observed during the hydrogen experiments could have been fueled by sulfur stores. For example, some *A. kojimai* –γ-1 holobionts incorporated inorganic carbon during treatments that did not supply any exogenous reductant. Based on the sulfur oxidation pathways used by the γ-1 symbiont [13, 14] and visual observation of the gill, the γ-1 symbiont deposits sulfur granules that could be accessed when exogenous reductants are insufficient. However, we did not observe upregulation of the DSR pathway, which would be expected if sulfur stores were mobilized during sulfide limitation [34, 35]. Interestingly, the γ-1 symbiont in association with *A. strummeri* upregulated genes of the SOX pathway, although no sulfidic reductants were added in the hydrogen treatment. Some marine thiotrophic *Gammaproteobacteria* possess an optimized SOX multi-enzyme system that has evolved to utilize trace amounts of thiosulfate resulting from the degradation of organic sulfur compounds [36]. If the *A. strummeri* symbiont has a similarly adapted system, it might have made use of host-derived sulfur-containing amino acids to generate thiosulfate as electron donor for chemosynthetic primary production.

In all experiments, the snails and their symbionts were not limited by oxygen concentration, since oxygen was present in the effluent. Still, the addition of nitrate during experimental treatment with both H_2_S and H_2_ stimulated the upregulation of genes for nitrate respiration by the γ-1 symbiont, likely to avoid competition for oxygen with its hosts [37, 38]. Although no such response was observed in the ε symbiont, genes for denitrification were present at moderate levels in its transcriptome, implying that it may use nitrate as alternative terminal electron acceptor for chemosynthesis.

## Conclusions

Our data reveal that the symbionts of *A. kojimai*, *A. strummeri*, and *A. boucheti* differ in their metabolic and transcriptomic responses to the supply of sulfide and hydrogen under controlled conditions. At the provided reductant concentrations, the γ-1 symbiont (associated with *A. kojimai* and *A. strummeri*) outperformed the ε symbiont (associated with *A. boucheti*) in terms of chemosynthetic activity. The ε symbiont fixed inorganic carbon when exposed to either hydrogen or sulfide, though at higher efficiency in the presence of hydrogen. Conversely, evidence for hydrogen-driven carbon fixation by the γ-1 symbiont is weak. Instead, the low rates of hydrogen oxidation, upregulation of sulfur oxidation genes in the H_2_ treatment, and demonstrated ability to fix inorganic carbon in the absence of an exogenous reductant, suggest that the γ-1 symbiont may be utilizing an endogenous energy source instead of or in addition to hydrogen when environmental sulfide is unavailable. These findings suggest that in hydrogen- and sulfide-deprived environments, the γ-1 symbiont may be more advantageous than the ε symbiont because of physiological adaptations to low reductant concentrations, which likely influence the observed host–symbiont niche differentiation along the ELSC. In the future, experiments with more replication and sampling times, longer acclimation and experimental periods, a greater range of hydrogen and sulfide concentrations, and the simultaneous application of both reductants, will be helpful to further inform our understanding of symbiont metabolic responses in the complete scope of conditions available to these holobionts. The work presented here provides initial insights into the physiological adaptations among *Alviniconcha* holobionts to local habitat conditions, and reinforces the hypothesis that diversity in chemoautotrophic traits may be an important driver of holobiont ecology at hydrothermal vents.

## Supplementary information

Supplementary Methods

Tables S1-7

Table S8

Table S9

Supplementary Figure Legends

Figure S1

Figure S2

Figure S3

Figure S4

Figure S5

Figure S6

Figure S7

Figure S8

## References

[1] McFall-Ngai M (2008). Are biologists in ‘future shock’? Symbiosis integrates biology across do-mains. Nat Rev Microbiol.

[2] McFall-Ngai M, Hadfield MG, Bosch TC, Carey HV, Domazet-Lošo T, Douglas AE (2013). Animals in a bacterial world, a new imperative for the life sciences. Proc Natl Acad Sci USA.

[3] Iglesias-Prieto R, Beltrán VH, LaJeunesse TC, Reyes-Bonilla H, Thomé PE (2004). Different algal symbionts explain the vertical distribution of dominant reef corals in the eastern Pacific. Proc Biol Sci.

[4] Bongaerts P, Frade PR, Ogier JJ, Hay KB, van Bleijswijk J, Englebert N (2013). Sharing the slope: depth partitioning of agariciid corals and associated *Symbiodinium* across shallow and mesophotic habitats (2-60 m) on a Caribbean reef. BMC Evol Biol.

[5] Joy JB (2013). Symbiosis catalyses niche expansion and diversification. Proc Biol Sci.

[6] Kohl KD, Carey HV (2016). A place for host-microbe symbiosis in the comparative physiologist’s toolbox. J Exp Biol.

[7] Minter EJA, Lowe CD, Sørensen MES, Wood AJ, Cameron DD, Brockhurst MA (2018). Variation and asymmetry in host-symbiont dependence in a microbial symbiosis. BMC Evol Biol.

[8] Dubilier N, Bergin C, Lott C (2008). Symbiotic diversity in marine animals: the art of harnessing chemosynthesis. Nat Rev Microbiol.

[9] Grassle JF (1985). Hydrothermal vent animals: distribution and biology. Science..

[10] Vrijenhoek RC (2010). Genetic diversity and connectivity of deep-sea hydrothermal vent metapopulations. Mol Ecol.

[11] Kim S, Hammerstrom K (2012). Hydrothermal vent community zonation along environmental gradients at the Lau back-arc spreading center. Deep-Sea Res I.

[12] Tivey MK (2007). Generation of seafloor hydrothermal vent fluids and associated mineral deposits. Oceanography.

[13] Beinart RA, Sanders JG, Faure B, Sylva SP, Lee RW, Becker EL (2012). Evidence for the role of endosymbionts in regional-scale habitat partitioning by hydrothermal vent symbioses. Proc Natl Acad Sci USA.

[14] Sanders JG, Beinart RA, Stewart FJ, Delong EF, Girguis PR (2013). Metatranscriptomics reveal differences in in situ energy and nitrogen metabolism among hydrothermal vent snail symbionts. ISMEJ..

[15] Johnson SB, Warén A, Tunnicliffe V, Van Dover C, Wheat CG, Schultz TF (2015). Molecular taxonomy and naming of five cryptic species of *Alviniconcha* snails (Gastropoda: Abyssochrysoidea) from hydrothermal vents. Syst Biodivers..

[16] Waite DW, Vanwonterghem I, Rinke C, Parks DH, Zhang Y, Takai K (2017). Comparative genomic analysis of the class Epsilonproteobacteria and proposed reclassification to Epsilonbacteraeota (phyl. nov.). Front Microbiol..

[17] Suzuki Y, Kojima S, Sasaki T, Suzuki M, Utsumi T, Watanabe H (2006). Host-symbiont relationships in hydrothermal vent gastropods of the genus *Alviniconcha* from the Southwest Pacific. Appl Environ Microbiol.

[18] Beinart RA, Luo C, Konstantinidis K, Stewart FJ, Girguis PR (2019). The bacterial symbionts of closely related hydrothermal vent snails with distinct geochemical habitats show broad similarity in chemoautotrophic gene content. Front Microbiol..

[19] Beinart RA, Gartman A, Sanders JG, Luther GW, Girguis PR (2015). The uptake and excretion of partially oxidized sulfur expands the repertoire of the energy resources metabolized by hydrothermal vent symbioses. Proc Biol Sci.

[20] Patro R, Duggal G, Love MI, Irizarry RA, Kingsford C (2017). Salmon provides fast and bias-aware quantification of transcript expression. Nat Methods..

[21] Grabherr MG, Haas BJ, Yassour M, Levin JZ, Thompson DA, Amit I (2011). Full-length transcriptome assembly from RNA-Seq data without a reference genome. Nat Biotechnol..

[22] Robinson MD, Oshlack A (2010). A scaling normalization method for differential expression analysis of RNA-seq data. Genome Biol..

[23] Love MI, Huber W, Anders S (2014). Moderated estimation of fold change and dispersion for RNA-seq data with DESeq2. Genome Biol..

[24] R Core Team. R: A language and environment for statistical computing. Vienna, Austria: R Foundation for Statistical Computing; 2018. URL https://www.R-project.org/.

[25] Podowski EL, Ma S, Luther GW, Wardrop D, Fisher CR (2010). Biotic and abiotic factors affecting distributions of megafauna in diffuse flow on andesite and basalt along the Eastern Lau Spreading Center, Tonga. Mar Ecol Prog Ser.

[26] Han Y, Perner M (2014). The role of hydrogen for *Sulfurimonas denitrificans*’ metabolism. PLoS ONE.

[27] McNichol J, Sievert SM (2019). Reconciling a model of core metabolism with growth yield predicts biochemical mechanisms and efficiency for a versatile chemoautotroph. bioRvix.

[28] van der Stel AX, Wösten MMSM (2019). Regulation of respiratory pathways in Campylobacterota: a review. Front Microbiol..

[29] Han Y, Perner M (2015). The globally widespread genus *Sulfurimonas*: versatile energy metabolisms and adaptations to redox clines. Front Microbiol..

[30] Fuchs G (2011). Alternative pathways of carbon dioxide fixation: insights into the early evolution of life?. Annu Rev Microbiol..

[31] Miyazaki J, Ikuta T, Watsuji TO, Abe M, Yamamoto M, Nakagawa S (2020). Dual energy metabolism of the Campylobacterota endosymbiont in the chemosynthetic snail *Alviniconcha marisindica*. ISMEJ..

[32] Yamamoto M, Nakagawa S, Shimamura S, Takai K, Horikoshi K (2010). Molecular characterization of inorganic sulfur-compound metabolism in the deep-sea epsilonproteobacterium Sulfurovum sp. NBC37-1. Environ Microbiol.

[33] Boyle NR, Morgan JA (2011). Computation of metabolic fluxes and efficiencies for biological carbon dioxide fixation. Metab Eng..

[34] Pott AS, Dahl C (1998). Sirohaem-sulfite reductase and other proteins encoded in the *dsr* locus of *Chromatium vinosum* are involved in the oxidation of intracellular sulfur. Microbiology.

[35] Dahl C, Engels S, Pott-Sperling AS, Schulte A, Sander J, Lübbe Y (2005). Novel genes of the *dsr* gene cluster and evidence for close interaction of Dsr proteins during sulfur oxidation in the phototrophic sulfur bacterium *Allochromatium vinosum*. J Bacteriol.

[36] Spring S (2014). Function and evolution of the Sox multienzyme complex in the marine gammaproteobacterium *Congregibacter litoralis*. ISRN Microbiol..

[37] Hentschel U, Hand S, Felbeck H (1996). The contribution of nitrate respiration to the energy budget of the symbiont-containing clam *Lucinoma aequizonata*: a calorimetric study. J Exp Biol.

[38] Roeselers G, Newton IL (2012). On the evolutionary ecology of symbioses between chemosynthetic bacteria and bivalves. Appl Microbiol Biotechnol.

