# Supplementary material for: Physiological dynamics of chemosynthetic symbionts in hydrothermal vent snails
Source: ISME J. 2020 Jul 2;14(10):2568–79. doi: 10.1038/s41396-020-0707-2 (PMC7490688; doi:10.1038/s41396-020-0707-2)
Supplement: Supplementary file 1 — Supplementary Methods [file 41396_2020_707_MOESM1_ESM.docx]

**Supplementary Methods**

*High-Pressure Respirometry System (HPRS)*

The HPRS was housed in a temperature-controlled intermodal shipping container maintained at 15–17°C, approximating the median temperature experienced by *Alviniconcha in situ* [1, 2]. Surface seawater from the ship’s metal-free seawater system was filtered through 0.2-μm inline cartridge filters (Millipore Inc.), UV-treated, and then pumped into a 200 L polypropylene storage and cooling tank. Seawater was subsequently pumped from the storage tank into an acrylic gas equilibration column, where it was supplied with carbon dioxide, oxygen, and nitrogen gas using mass flow controllers (Aalborg Instruments and Controls, Inc.) to achieve concentrations of 4 mM, >250 µM, and 400 µM, respectively. Depending on the experiment, hydrogen or hydrogen sulfide gas was also supplied via mass flow controllers (Aalborg Instruments and Controls, Inc.) to achieve concentrations of approximately ~25 µM and ~120 µM, respectively. In addition, sodium nitrate (NaNO_3_) was added to achieve a final concentration of 40 µM, which is comparable to deep-ocean water. The pH of the resulting input water was 6–7. Seawater from the equilibration column was then supplied to three high-pressure metering pumps (Lewa GmbH) equipped with titanium wetted parts. The pumps delivered fluid into three titanium high-pressure aquaria at a rate of 20 ml min^-1^. Pressure was maintained via 316 stainless steel backpressure valves (StraVal Inc). Input water and effluent samples were taken every four hours for monitoring of pH using a handheld pH meter.

*Reductant and oxygen concentrations*

Every 4 hours, 2 ml of input or effluent water was preserved with a 2 mM zinc acetate solution and frozen at –80ºC until analysis as in [3]. At the same timepoints, dissolved H_2_ was analyzed at sea by gas chromatography using a 5Å molecular sieve packed column and thermal conductivity detection after headspace extraction in a gas-tight glass syringe. Oxygen concentrations were continuously monitored with the Oxy-4 SMA multi-channel optical oxygen meter and flow-through cell sensors FTC-SU-Pst3 (PreSens Precision Sensing, GmbH).

*Empty and estimated controls*

Some loss of both hydrogen and sulfide was observed in the control aquaria devoid of snails, which equaled ~32 to 37% of the reductant used by the snails. These loss rates in our controls are likely due to unexpected reactivity with metals within the control vessels or diffusional loss from the system, and not due to depletion by free-living microbes, because dissolved oxygen concentrations did not change proportionally. Nevertheless, this loss is insufficient to account for the observed differences in holobiont uptake and, therefore, does not likely explain the observed differences in reductant utilization between each holobiont.

*Mitochondrial COI gene and transcriptomic sequencing*

Gill tissue pieces were excised from each animal and homogenized in TRIzol™ reagent (Thermo Fisher Scientific, Inc.) using a Tissue-Tearor (Biospec Products, Inc.). Additionally, gill tissue pieces from all individuals were preserved in RNALater™. All samples were stored at –80ºC until RNA and DNA extraction with the Direct-zol RNA and Quick-DNA 96 Plus extraction kits (Zymo Research, Inc.), respectively. Species identities were confirmed by their mitochondrial *COI* gene signatures as in [4]. Transcriptomic sequencing libraries were created with the RNAtag-Seq approach [5] after rRNA depletion, and then sequenced with a 2 x 33–75 bp paired-end protocol on Next- and NovaSeq (Illumina, Inc.) instruments at the Broad Institute Microbial ‘Omics Core (Cambridge, MA, USA).

*Bioinformatic analyses*

Raw sequence reads were quality checked with FASTQC [6] and then trimmed with TRIMMOMATIC [7] using all built-in adaptor sequences and the following clipping settings: ILLUMINACLIP:Illumina.fa:2:30:10 SLIDINGWINDOW:4:20 LEADING:5 TRAILING:5 MINLEN:25. To remove potential sequence contaminants the trimmed reads were aligned against the human and PhiX genomes with BOWTIE2 [8]. All unmapped paired-end reads were extracted with SEQTK (<https://github.com/lh3/seqtk>). Ribosomal sequence removal in SORTMERNA [9] was done with default settings except that we excluded paired-end reads where only one mate aligned to the rRNA databases (i.e., --paired_in option). BBSPLIT (<https://sourceforge.net/projects/bbmap/>) was applied to sort host and symbiont sequences by mapping the reads against draft genomes of the three *campylobacterial* and *gammaproteobacterial* *Alviniconcha* symbiont phylotypes [10]. Reads with ambiguous alignments were discarded. Sequencing of *A. boucheti* #79 from Experiment 3 failed and this sample was therefore excluded from further analyses. Read quantification was done in SALMON [11] with the following parameters: -l ISR --meta --rangeFactorizationBins 4 --numBootstraps 1000 --seqBias --gcBias -s -u. Differential gene expression was analyzed with *DESeq2* in R [12, 13]. To account for batch effects and pseudo-replication we grouped all snail specimens according to tank and included this factor into the model design. We further corrected for variability in animal weight and time to dissection after depressurization. P-values were adjusted for type I error with the Benjamini-Hochberg method [14] at a significance threshold of 0.05. Heatmaps, PCA plots and Likert graphs were generated in R using the *DESeq2*, *gplots* and *HH* packages [12, 15, 16].

**References**

1. Podowski EL, Ma S, Luther GW III, Wardrop D, Fisher CR. Biotic and abiotic factors affecting distributions of megafauna in diffuse flow on andesite and basalt along the Eastern Lau Spreading Center, Tonga. Mar Ecol Prog Ser. 2010; 418: 25–45.
2. Beinart RA, Sanders JG, Faure B, Sylva SP, Lee RW, Becker EL *et al.* Evidence for the role of endosymbionts in regional-scale habitat partitioning by hydrothermal vent symbioses. PNAS 2012; 109: E3241–3250.
3. Mitchell JH, Leonard JM, Delaney J, Girguis PR, Scott KM. Hydrogen does not appear to be a major electron donor for the deep-sea hydrothermal vent symbiosis *Riftia pachyptila*. Appl Environ Microbiol. 2019; doi: 10.1128/AEM.01522-19.
4. Johnson SB, Warén A, Tunnicliffe V, Van Dover C, Wheat CG, Schultz TF *et al.* Molecular taxonomy and naming of five cryptic species of *Alviniconcha* snails (Gastropoda: Abyssochrysoidea) from hydrothermal vents. Syst Biodivers. 2015; 13: 278–295.
5. Shishkin AA, Giannoukos G, Kucukural A, Ciulla D, Busby M, Surka C *et al.* Simultaneous generation of many RNA-seq libraries in a single reaction. Nat Methods. 2015; 12: 323–325.
6. Andrews S. FastQC: a quality control tool for high throughput sequence data. <http://www.bioinformatics.babraham.ac.uk/projects/fastqc/> (2010)
7. Bolger AM, Lohse M, Usadel B. Trimmomatic: a flexible trimmer for Illumina sequence data. Bioinformatics 2014; 30: 2114–2120.
8. Langmead B, Salzberg SL. Fast gapped-read alignment with Bowtie 2. Nat Methods. 2012; 9: 357–359.
9. Kopylova E, Noé L, Touzet H. SortMeRNA: fast and accurate filtering of ribosomal RNAs in metatranscriptomic data. Bioinformatics 2012; 28: 3211–3217.
10. Beinart RA, Luo C, Konstantinidis K, Stewart FJ, Girguis PR. The bacterial symbionts of closely related hydrothermal vent snails with distinct geochemical habitats show broad similarity in chemoautotrophic gene content. Front Microbiol. 2019; 10: 1818.
11. Patro R, Duggal G, Love MI, Irizarry RA, Kingsford C. Salmon provides fast and bias-aware quantification of transcript expression. Nat Methods. 2017; 14: 417–419.
12. Love MI, Huber W, Anders S. Moderated estimation of fold change and dispersion for RNA-seq data with DESeq2. Genome Biol. 2014; 15: 550.
13. R Core Team. R: A language and environment for statistical computing. R Foundation for Statistical Computing, Vienna, Austria. 2018. URL https://www.R-project.org/.
14. Benjamini Y, Hochberg Y. Controlling the false discovery rate: a practical and powerful approach to multiple testing. J R Statist Soc B 1995; 57: 289–300.
15. Heiberger RM, Robbins NB. Design of diverging stacked bar charts for Likert scales and other applications. J Statist Softw 2014; 57: 1–32.
16. Warnes GR, Bolker B, Bonebakker L, Gentleman R, Huber W, Liaw A et al. gplots: various R programming tools for plotting data. R package version 3.0.1.1 2019.
