# Supplementary material for: Physiological dynamics of chemosynthetic symbionts in hydrothermal vent snails
Source: ISME J. 2020 Jul 2;14(10):2568–79. doi: 10.1038/s41396-020-0707-2 (PMC7490688; doi:10.1038/s41396-020-0707-2)
Supplement: Supplementary file 2 — Tables S1-7 [file 41396_2020_707_MOESM2_ESM.docx]

**Table S1** *In situ* concentrations of H_2_S and O_2_ as well as thermal conditions in diffuse flow habitats at the investigated vent sites. *In situ* H_2_ values represent end-member concentrations at high-temperature orifices, as diffuse flow H_2_ concentrations have not been measured at these sites. Measurements from Tow Cam, ABE, and Tu’i Malila were made in 2009 (Beinart et al. 2012) and from Tahi Moana in 2016 (J. Seewald, unpublished data).

| **Vent field** | **H_2_S [µM]** | **H_2_ [µM]** | **O_2_ [µM]** | **Temperature [°C]** |
| --- | --- | --- | --- | --- |
| Tow Cam | 66.6–75.0 | 100.0–200.0 | 2.5 | 14.0–17.2 |
| Tahi Moana | – | 97 | – | – |
| ABE | 38.8–356.0 | 50.0–100.0 | 2.5–45.0 | 9.2–60.2 |
| Tu’i Malila | 7.9–49.5 | 35.0–135.0 | 2.5–71.0 | 10.9–27.3 |

**Table S2** Summary of H_2_S and H_2_ concentrations in input water, empty control aquaria, effluent water of aquaria containing *Alviniconcha*, and the calculated mass-specific mean oxidation rates with standard deviations (SD) and standard errors (SE).

| **Exp.** | **Reductant** | **Species** | **Total gill weight (g)** | **Input water mean (µM)** | **Empty control mean (µM)** | **Effluent water mean (µM)** | **Oxidation rate**  **(µmoles g^-1^ hr^-1^)** | **SD** | **SE** | **Time to steady state [h]^*^** |
| --- | --- | --- | --- | --- | --- | --- | --- | --- | --- | --- |
| 1 | H_2_S | *A. boucheti* | 30.80 | 117.67 | ^†^80.02 | 0.00 | 3.12 | 0.11 | 0.05 | 12 |
| 1 | H_2_S | *A. kojimai* | 18.30 | 117.67 | ^†^80.02 | 0.00 | 5.26 | 0.19 | 0.08 | 12 |
| 2 | H_2_S | *A. kojimai* | 11.40 | 122.43 | ^†^83.25 | 0.17 | 8.74 | 0.36 | 0.16 | 8 |
| 2 | H_2_S | *A. strummeri* | 8.50 | 122.43 | ^†^83.25 | 3.62 | 11.24 | 0.46 | 0.21 | 8 |
| 3 | H_2_ | *A. strummeri* | 13.20 | 26.40 | ^‡^16.63 | 18.29 | 0.18 | 0.68 | 0.30 | 8 |
| 3 | H_2_ | *A. kojimai* | 6.90 | 26.40 | ^‡^16.63 | 15.71 | 0.16 | 0.22 | 0.10 | 8 |
| 4 | H_2_ | *A. boucheti* | 8.80 | 27.34 | 21.29 | 5.30 | 2.18 | 0.69 | 0.28 | 4 |
| 4 | H_2_ | *A. kojimai* | 19.00 | 27.34 | 21.29 | 1.46 | 1.25 | 0.26 | 0.11 | 4 |
| 5 | H_2_ | *A. strummeri* | 35.10 | 26.09 | 13.52 | 6.68 | 0.23 | 0.09 | 0.03 | 4 |
| 5 | H_2_ | *A. boucheti* | 20.70 | 26.09 | 13.52 | 0.15 | 0.78 | 0.08 | 0.03 | 4 |
| Control | H_2_S | N/A | N/A | 146.16 | 99.80 | N/A | N/A | N/A | N/A | 8 |

^*^Time of calculation start

^†^Empty control values were estimated with a 0.68 correction factor applied to the input water concentration

^‡^Empty control values were estimated with a 0.63 correction factor applied to the input water concentration

**Table S3** Summary of oxygen concentrations in input, empty control aquaria, effluent water of aquaria containing *Alviniconcha*, and the calculated mass-specific mean respiration rates with standard deviations (SD) and standard errors (SE).

| **Exp.** | **Species** | **Total gill weight (g)** | **Input water mean (µM)** | **Empty control mean (µM)** | **Effluent water mean (µM)** | **Respiration rate**  **(µmoles g^-1^ hr^-1^)** | **SD** | **SE** | **Time to steady state [h]^*^** |
| --- | --- | --- | --- | --- | --- | --- | --- | --- | --- |
| 1 | *A. boucheti* | 30.80 | 231.77 | ^†^250.31 | 7.45 | 9.34 | 0.28 | 0.02 | 4 |
| 1 | *A. kojimai* | 18.30 | 231.77 | ^†^250.31 | 10.63 | 15.72 | 0.47 | 0.03 | 4 |
| 2 | *A. kojimai* | 11.40 | 232.50 | ^†^251.10 | 11.80 | 23.45 | 1.40 | 0.10 | 4 |
| 2 | *A. strummeri* | 8.50 | 232.50 | ^†^251.10 | 28.33 | 31.45 | 1.87 | 0.14 | 4 |
| 3 | *A. strummeri* | 13.20 | 254.81 | ^‡^285.39 | 169.37 | 7.65 | 0.62 | 0.06 | 4 |
| 3 | *A. kojimai* | 6.90 | 254.81 | ^‡^285.39 | 201.25 | 14.63 | 1.18 | 0.11 | 4 |
| 4 | *A. boucheti* | 8.80 | 259.06 | 282.44 | 141.74 | 35.19 | 2.26 | 0.30 | 4 |
| 4 | *A. kojimai* | 19.00 | 259.06 | 282.44 | 24.38 | 16.30 | 1.05 | 0.14 | 4 |
| 5 | *A. strummeri* | 35.10 | 258.19 | 294.71 | 82.28 | 9.41 | 0.20 | 0.01 | 4 |
| 5 | *A. boucheti* | 20.70 | 258.19 | 294.71 | 19.60 | 15.95 | 0.33 | 0.02 | 4 |
| Control | N/A | N/A | 257.99 | 277.73 | N/A | N/A | N/A | N/A | 4 |

^*^Time of calculation start

^†^Empty control values were estimated with a 1.08 correction factor relative to the input concentration

^‡^Empty control values were estimated with a 1.12 correction factor relative to the input concentration

**Table S4** Representation of *Alviniconcha* symbiont phylotypes in each host individual. The table shows the number of transcripts for each phylotype. Percent proportions are given in brackets.

| **Exp.** | **Individual** | **Species** | **Group** | **ε reads (%)** | **γ-1 reads (%)** | **γ-Lau reads (%)** |
| --- | --- | --- | --- | --- | --- | --- |
| 1 | 01 | *A. boucheti* | Control | 3 581 914 (81.20) | 808 477 (18.33) | 21 016 (0.48) |
| 1 | 02 | *A. boucheti* | Control | 2 248 625 (78.15) | 612 650 (21.29) | 16 006 (0.56) |
| 1 | 03 | *A. kojimai* | Control | 1 037 241 (21.80) | 3 602 363 (75.70) | 119 360 (2.51) |
| 1 | 04 | *A. kojimai* | Control | 905 981 (20.75) | 3 379 806 (77.40) | 80 816 (1.85) |
| 1 | 05 | *A. boucheti* | Treatment | 2 461 355 (81.33) | 547 412 (18.09) | 17 581 (0.58) |
| 1 | 06 | *A. boucheti* | Treatment | 2 782 584 (73.21) | 998 166 (26.26) | 20 024 (0.53) |
| 1 | 07 | *A. boucheti* | Treatment | 4 220 319 (85.84) | 678 021 (13.79) | 18 439 (0.38) |
| 1 | 08 | *A. boucheti* | Treatment | 915 371 (68.45) | 413 707 (30.94) | 8 126 (0.61) |
| 1 | 09 | *A. kojimai* | Treatment | 1 184 644 (18.31) | 5 149 243 (79.59) | 136 054 (2.10) |
| 1 | 10 | *A. kojimai* | Treatment | 1 683 024 (16.38) | 8 348 230 (81.26) | 242 548 (2.36) |
| 1 | 11 | *A. kojimai* | Treatment | 1 402 207 (18.84) | 5 910 237 (79.40) | 131 528 (1.77) |
| 1 | 12 | *A. kojimai* | Treatment | 2 596 042 (18.12) | 11 392 363 (79.51) | 340 486 (2.38) |
| 2 | 01 | *A. kojimai* | Control | 1 470 943 (23.55) | 4 673 259 (74.81) | 102 265 (1.64) |
| 2 | 02 | *A. strummeri* | Control | 1 415 371 (17.97) | 6 286 691 (79.83) | 173 361 (2.20) |
| 2 | 03 | *A. kojimai* | Control | 1 368 303 (17.16) | 5 866 948 (73.59) | 737 095 (9.25) |
| 2 | 04 | *A. strummeri* | Control | 1 329 001 (20.55) | 4 922 372 (76.13) | 214 354 (3.32) |
| 2 | 05 | *A. strummeri* | Control | 1 838 037 (19.37) | 7 375 268 (77.71) | 278 012 (2.93) |
| 2 | 06 | *A. kojimai* | Control | 1 674 319 (17.53) | 7 377 506 (77.23) | 500 221 (5.24) |
| 2 | 07 | *A. strummeri* | Treatment | 1 302 966 (15.43) | 6 877 732 (81.44) | 264 658 (3.13) |
| 2 | 08 | *A. strummeri* | Treatment | 1 182 693 (15.68) | 6 201 940 (82.21) | 159 124 (2.11) |
| 2 | 09 | *A. strummeri* | Treatment | 1 359 903 (18.26) | 5 842 009 (78.42) | 247 556 (3.32) |
| 2 | 11 | *A. kojimai* | Treatment | 1 229 324 (13.49) | 7 727 309 (84.77) | 158 737 (1.74) |
| 2 | 12 | *A. kojimai* | Treatment | 1 306 221 (12.15) | 9 247 371 (85.99) | 200 708 (1.87) |
| 2 | 13 | *A. kojimai* | Treatment | 2 875 825 (12.55) | 19 636 985 (85.70) | 400 284 (1.75) |
| 3 | 02 | *A. kojimai* | Control | 1 597 507 (17.65) | 7 289 767 (80.54) | 163 806 (1.81) |
| 3 | 03 | *A. strummeri* | Control | 1 843 238 (16.19) | 9 222 744 (81.01) | 318 027 (2.79) |
| 3 | 04 | *A. strummeri* | Control | 3 350 367 (14.75) | 18 414 432 (81.07) | 950 515 (4.18) |
| 3 | 05 | *A. kojimai* | Control | 1 666 455 (17.79) | 7 542 980 (80.52) | 157 993 (1.69) |
| 3 | 06 | *A. kojimai* | Control | 1 570 563 (15.87) | 7 701 626 (77.81) | 626 075 (6.33) |
| 3 | 07 | *A. strummeri* | Treatment | 1 534 067 (31.55) | 3 026 610 (62.24) | 301 883 (6.21) |
| 3 | 08 | *A. strummeri* | Treatment | 1 322 654 (24.36) | 3 929 230 (72.36) | 177 920 (3.28) |
| 3 | 09 | *A. strummeri* | Treatment | 2 484 455 (28.62) | 5 630 235 (64.86) | 566 347 (6.52) |
| 3 | 11 | *A. kojimai* | Treatment | 2 267 843 (24.74) | 6 707 837 (73.18) | 190 233 (2.08) |
| 3 | 12 | *A. kojimai* | Treatment | 1 476 152 (24.16) | 4 529 377 (74.15) | 103 229 (1.69) |
| 3 | 13 | *A. kojimai* | Treatment | 1 438 625 (18.94) | 6 016 300 (79.20) | 141 208 (1.86) |
| 4 | 01 | *A. boucheti* | Control | 2 686 415 (74.44) | 900 919 (24.96) | 21 541 (0.60) |
| 4 | 02 | *A. kojimai* | Control | 2 179 563 (23.73) | 6 845 617 (74.52) | 161 362 (1.76) |
| 4 | 03 | *A. kojimai* | Control | 970 151 (20.48) | 3 675 895 (77.61) | 90 586 (1.91) |
| 4 | 04 | *A. boucheti* | Control | 2 544 034 (71.50) | 991 746 (27.87) | 22 466 (0.63) |
| 4 | 05 | *A. boucheti* | Treatment | 1 803 962 (76.68) | 536 083 (22.79) | 12 512 (0.53) |
| 4 | 06 | *A. boucheti* | Treatment | 2 662 522 (65.65) | 1 364 527 (33.65) | 28 520 (0.70) |
| 4 | 07 | *A. boucheti* | Treatment | 2 788 583 (73.36) | 991 807 (26.09) | 20 914 (0.55) |
| 4 | 08 | *A. boucheti* | Treatment | 2 536 310 (76.79) | 750 169 (22.71) | 16 484 (0.50) |
| 4 | 09 | *A. kojimai* | Treatment | 1 404 908 (18.99) | 5 876 214 (79.41) | 118 953 (1.61) |
| 4 | 10 | *A. kojimai* | Treatment | 1 488 474 (30.78) | 3 278 902 (67.81) | 68 028 (1.41) |
| 4 | 11 | *A. kojimai* | Treatment | 1 200 072 (20.33) | 4 603 081 (77.96) | 100 992 (1.71) |
| 4 | 12 | *A. kojimai* | Treatment | 1 415 543 (25.92) | 3 952 525 (72.39) | 92 200 (1.69) |
| 5 | 01 | *A. boucheti* | Control | 3 524 284 (75.64) | 1 110 310 (23.83) | 24 780 (0.53) |
| 5 | 02 | *A. boucheti* | Control | 3 328 057 (80.90) | 768 212 (18.67) | 17 546 (0.43) |
| 5 | 03 | *A. boucheti* | Control | 4 768 166 (81.62) | 1 047 155 (17.92) | 26 701 (0.46) |
| 5 | 04 | *A. strummeri* | Control | 1 453 041 (27.26) | 3 633 658 (68.18) | 242 740 (4.55) |
| 5 | 05 | *A. strummeri* | Control | 2 224 965 (17.84) | 9 678 874 (77.63) | 564 674 (4.53) |
| 5 | 06 | *A. strummeri* | Control | 1 638 430 (19.31) | 6 458 763 (76.12) | 388 318 (4.58) |
| 5 | 07 | *A. strummeri* | Treatment | 1 623 083 (28.91) | 3 147 097 (56.06) | 843 441 (15.02) |
| 5 | 08 | *A. strummeri* | Treatment | 1 738 641 (31.97) | 3 407 056 (62.65) | 292 685 (5.38) |
| 5 | 09 | *A. strummeri* | Treatment | 2 035 689 (32.58) | 3 736 265 (59.80) | 475 905 (7.62) |
| 5 | 10 | *A. boucheti* | Treatment | 2 821 060 (78.33) | 759 122 (21.08) | 21 301 (0.59) |
| 5 | 11 | *A. boucheti* | Treatment | 2 709 549 (74.55) | 906 725 (24.95) | 18 116 (0.50) |
| 5 | 12 | *A. boucheti* | Treatment | 1 040 013 (78.27) | 283 494 (21.34) | 5 197 (0.39) |

**Table S5** δ^13^C and A% means and standard deviations (SD) for gill tissue from acclimation individuals from each experiment. Also shown is the enrichment threshold used for each experiment, which is the acclimation gill A% mean plus two times the standard deviation.

| **Experiment** | **Species** | **Mean δ^13^C** | **SD δ^13^C** | **Mean A%** | **SD A%** | **Enrichment threshold (A% mean + 2*SD)** |
| --- | --- | --- | --- | --- | --- | --- |
| 1 | *A. boucheti* | –12.39 | 0.10 | 1.09205166 | 0.00010828 | 1.09226822 |
| 1 | *A. kojimai* | –30.73 | 0.37 | 1.07198944 | 0.00041065 | 1.07281074 |
| 2 | *A. kojimai* | –29.32 | 0.28 | 1.07353111 | 0.00030746 | 1.07414602 |
| 2 | *A. strummeri* | –29.48 | 0.13 | 1.07336110 | 0.00014353 | 1.07364815 |
| 3 | *A. kojimai* | –29.56 | 0.25 | 1.07326739 | 0.00027382 | 1.07381503 |
| 3 | *A. strummeri* | –29.54 | 0.15 | 1.07328813 | 0.00016478 | 1.07361770 |
| 4 | *A. boucheti* | –12.37 | 0.21 | 1.09207638 | 0.00022952 | 1.09253542 |
| 4 | *A. kojimai* | –30.95 | 0.18 | 1.07174672 | 0.00019975 | 1.07214623 |
| 5 | *A. boucheti* | –11.23 | 0.07 | 1.09332089 | 0.00007085 | 1.09346259 |
| 5 | *A. strummeri* | –30.20 | 0.21 | 1.07256748 | 0.00022726 | 1.07302200 |

**Table S6** Summary of isotopic labeling and carbon fixation rates. The table shows the δ^13^C and A% of each gill tissue sample, the A% of the dissolved inorganic carbon (DIC) supplied in the experiment, the percent carbon in each gill tissue sample (%C), the dry:wet tissue ratio for each sample, the duration of each experiment, and the resulting mass-specific rate of inorganic carbon incorporation for each individual (^13^C_inc_).

| **Exp.** | **Snail ID** | **Species** | **Gill weight (g)** | **δ^13^C** | **A%** | **DIC A%** | **%C** | **Dry:wet** | **Duration (h)** | **^13^C_inc_**  **(µmoles g^-1^ hr^-1^)** |
| --- | --- | --- | --- | --- | --- | --- | --- | --- | --- | --- |
| 1 | 05 | *A. boucheti* | 8.7543 | –10.52 | 1.094092258 | 3.299660437 | 44.77 | ^*^0.74 | 28.00 | 1.18 |
| 1 | 06 | *A. boucheti* | 6.0953 | –11.16 | 1.093396064 | 3.299660437 | 43.67 | ^*^0.74 | 28.00 | 0.76 |
| 1 | 07 | *A. boucheti* | 7.1625 | –11.35 | 1.093188673 | 3.299660437 | 45.51 | ^*^0.74 | 28.00 | 0.67 |
| 1 | 08 | *A. boucheti* | 8.7815 | –11.69 | 1.092813364 | 3.299660437 | 45.70 | ^*^0.74 | 28.00 | 0.45 |
| 1 | 09 | *A. kojimai* | 2.5635 | –11.83 | 1.092660695 | 3.299660437 | 40.15 | 0.90 | 28.50 | 8.53 |
| 1 | 10 | *A. kojimai* | 6.5878 | –2.97 | 1.108843771 | 3.299660437 | 40.89 | 0.82 | 28.50 | 16.99 |
| 1 | 11 | *A. kojimai* | 6.5432 | –27.76 | 1.075243610 | 3.299660437 | 39.30 | 0.79 | 28.50 | 1.50 |
| 1 | 12 | *A. kojimai* | 2.5635 | –24.33 | 1.078994479 | 3.299660437 | 36.27 | 0.88 | 28.50 | 2.67 |
| 2 | 10 | *A. kojimai* | 1.6880 | –21.00 | 1.082629253 | 2.527182316 | 37.09 | 0.80 | 23.00 | 7.41 |
| 2 | 11 | *A. kojimai* | 3.0172 | –23.13 | 1.080307728 | 2.527182316 | 34.40 | 0.74 | 23.00 | 5.53 |
| 2 | 12 | *A. kojimai* | 3.7959 | –22.65 | 1.080831912 | 2.527182316 | 39.82 | 0.85 | 23.00 | 6.01 |
| 2 | 13 | *A. kojimai* | 2.6517 | –23.51 | 1.079886769 | 2.527182316 | 38.89 | 0.75 | 23.00 | 5.79 |
| 2 | 14 | *A. kojimai* | 1.9464 | –22.15 | 1.081379019 | 2.527182316 | 37.36 | 0.68 | 23.00 | 7.57 |
| 2 | 07 | *A. strummeri* | 2.2371 | –24.52 | 1.078783447 | 2.527182316 | 37.91 | 0.74 | 23.50 | 4.77 |
| 2 | 08 | *A. strummeri* | 2.0991 | –24.16 | 1.079180439 | 2.527182316 | 38.23 | 0.73 | 23.50 | 5.24 |
| 2 | 09 | *A. strummeri* | 2.4715 | –28.94 | 1.073947398 | 2.527182316 | 39.03 | 0.80 | 23.50 | 0.49 |
| 3 | 11 | *A. kojimai* | 1.6613 | –28.85 | 1.074043593 | 4.000878135 | 54.56 | 0.79 | 25.38 | 0.42 |
| 3 | 12 | *A. kojimai* | 1.7772 | –27.18 | 1.075868643 | 4.000878135 | 55.63 | 0.87 | 25.38 | 1.31 |
| 3 | 13 | *A. kojimai* | 1.3377 | –27.20 | 1.075852807 | 4.000878135 | 56.71 | 0.79 | 25.38 | 1.47 |
| 3 | 14 | *A. kojimai* | 1.2364 | –27.45 | 1.075580133 | 4.000878135 | 57.78 | 0.84 | 25.38 | 1.26 |
| 3 | 15 | *A. kojimai* | 0.8901 | –27.50 | 1.075519913 | 4.000878135 | 58.86 | 0.93 | 25.38 | 1.13 |
| 3 | 07 | *A. strummeri* | 2.5274 | –28.68 | 1.074236604 | 4.000878135 | 50.26 | 0.81 | 24.73 | 0.48 |
| 3 | 08 | *A. strummeri* | 2.5274 | –28.38 | 1.074563638 | 4.000878135 | 51.34 | 0.68 | 24.73 | 0.78 |
| 3 | 09 | *A. strummeri* | 2.9292 | –28.56 | 1.074364966 | 4.000878135 | 52.41 | 0.87 | 24.73 | 0.53 |
| 3 | 10 | *A. strummeri* | 1.1823 | –28.66 | 1.074253890 | 4.000878135 | 53.49 | 0.85 | 24.73 | 0.49 |
| 4 | 05 | *A. boucheti* | 1.7879 | –11.13 | 1.093424771 | 4.127155032 | 64.23 | 0.83 | 22.97 | 0.88 |
| 4 | 06 | *A. boucheti* | 2.9194 | –10.76 | 1.093835095 | 4.127155032 | 65.30 | 0.70 | 22.97 | 1.38 |
| 4 | 07 | *A. boucheti* | 2.2062 | –11.45 | 1.093084552 | 4.127155032 | 66.37 | 0.78 | 22.97 | 0.72 |
| 4 | 08 | *A. boucheti* | 1.8867 | –10.61 | 1.093992763 | 4.127155032 | 67.45 | 0.79 | 22.97 | 1.38 |
| 4 | 09 | *A. kojimai* | 5.2610 | –24.38 | 1.078939319 | 4.127155032 | 68.52 | 0.62 | 23.43 | 6.52 |
| 4 | 10 | *A. kojimai* | 4.4537 | –28.06 | 1.074912478 | 4.127155032 | 69.60 | 0.55 | 23.43 | 3.28 |
| 4 | 11 | *A. kojimai* | 4.3012 | –12.97 | 1.091420600 | 4.127155032 | 70.67 | 0.57 | 23.43 | 20.00 |
| 4 | 12 | *A. kojimai* | 5.0063 | –27.85 | 1.075144518 | 4.127155032 | 71.74 | 0.60 | 23.43 | 3.33 |
| 5 | 10 | *A. boucheti* | 8.8658 | –9.58 | 1.095120717 | 3.519733138 | 80.34 | 0.58 | 37.88 | 1.59 |
| 5 | 11 | *A. boucheti* | 5.7685 | –9.71 | 1.094985002 | 3.519733138 | 80.87 | 0.60 | 37.88 | 1.43 |
| 5 | 12 | *A. boucheti* | 6.0638 | –9.75 | 1.094936538 | 3.519733138 | 81.41 | 0.70 | 37.88 | 1.20 |
| 5 | 07 | *A. strummeri* | 10.6778 | –26.59 | 1.076520375 | 3.519733138 | 78.19 | 0.58 | 36.83 | 3.47 |
| 5 | 08 | *A. strummeri* | 9.5858 | –27.37 | 1.075668104 | 3.519733138 | 78.72 | 0.71 | 36.83 | 2.24 |
| 5 | 09 | *A. strummeri* | 14.8120 | –28.76 | 1.074148973 | 3.519733138 | 79.26 | 0.58 | 36.83 | 1.41 |
| 6 | 01 | *A. kojimai* | 5.4844 | –29.12 | 1.073753179 | 3.428500992 | 82.48 | 0.69 | 25.30 | NA |
| 6 | 02 | *A. kojimai* | 2.8754 | –27.86 | 1.075126947 | 3.428500992 | 83.56 | 0.66 | 25.30 | NA |
| 6 | 03 | *A. kojimai* | 7.5454 | –24.40 | 1.078912252 | 3.428500992 | 84.63 | 0.64 | 25.30 | NA |
| 6 | 04 | *A. boucheti* | 3.6659 | –12.27 | 1.092177102 | 3.428500992 | 85.71 | 0.74 | 24.85 | NA |
| 6 | 05 | *A. boucheti* | 4.1800 | –11.46 | 1.093069909 | 3.428500992 | 86.78 | 0.73 | 24.85 | NA |
| 6 | 06 | *A. boucheti* | 5.3712 | –12.82 | 1.091577939 | 3.428500992 | 87.85 | 0.59 | 24.85 | NA |

^*^Average values were used because weights were missing

**Table S7** Transcript proportional abundances (%) for selected key genes related to sulfur, hydrogen, nitrogen and carbon metabolism. Sox = Sox multienzyme complex; Sat = sulfur adenylyltransferase; AprAB = adenylylsulfate reductase; DsrAB = (reverse) dissimilatory sulfite reductase; Sqr = sulfide quinone oxidoreductase; HyaAB = uptake [NiFe] hydrogenase; Nap = periplasmic nitrate reductase; NarK = nitrate/nitrite transporter; NirA = ferredoxin-dependent nitrite reductase; NirBD = NAD(P)H-dependent siroheme nitrite reductase; NirS = membrane-bound respiratory nitrite reductase; NorBC = nitric oxide reductase; NosZ = nitrous oxide reductase; GS+=GOGAT = glutamine synthetase + glutamate synthase; CBB = Calvin-Benson-Bassham cycle; rTCA = reverse tricarboxylic acid cycle.

|  |  | ***A. boucheti* – ε** | | | ***A. kojimai* – γ-1** | | | ***A. strummeri* –** **γ-1** | | |
| --- | --- | --- | --- | --- | --- | --- | --- | --- | --- | --- |
| **Metabolism** | **Gene** | **Control** | **H_2_S** | **H_2_** | **Control** | **H_2_S** | **H_2_** | **Control** | **H_2_S** | **H_2_** |
| Sulfur | SoxABXYZ | 1.25 | 0.71 | 1.92 | 0.32 | 0.83 | 0.63 | 0.65 | 0.84 | 1.29 |
|  | SoxCD | 1.38 | 0.99 | 1.06 | – | – | – | – | – | – |
|  | Sat | 0.02 | 0.02 | 0.01 | 0.06 | 0.12 | 0.05 | 0.07 | 0.16 | 0.03 |
|  | AprAB | – | – | – | 0.39 | 0.65 | 0.43 | 0.33 | 0.68 | 0.12 |
|  | DsrAB | – | – | – | 0.44 | 0.60 | 0.35 | 0.30 | 0.52 | 0.11 |
|  | Sqr | 0.40 | 0.30 | 0.33 | 0.11 | 0.21 | 0.09 | 0.16 | 0.26 | 0.06 |
|  | **Total** | **3.05** | **2.02** | **3.32** | **1.32** | **2.41** | **1.55** | **1.51** | **2.46** | **1.61** |
| Hydrogen | HyaAB | 0.02 | 0.04 | 0.02 | 0.00 | 0.01 | 0.01 | 0.01 | 0.01 | 0.00 |
| Nitrogen | NapABGH | 1.93 | 1.13 | 1.44 | 0.25 | 0.29 | 0.13 | 0.30 | 0.22 | 0.15 |
|  | NapC | – | – | – | 0.07 | 0.22 | 0.05 | 0.07 | 0.18 | 0.03 |
|  | NarK | 0.04 | 0.03 | 0.04 | 0.01 | 0.03 | 0.01 | 0.02 | 0.07 | 0.01 |
|  | NirA | 0.03 | 0.02 | 0.02 | – | – | – | – | – | – |
|  | NirBD | – | – | – | 0.01 | 0.04 | 0.01 | 0.02 | 0.07 | 0.01 |
|  | NirS | 1.53 | 0.89 | 1.24 | 0.04 | 0.06 | 0.04 | 0.02 | 0.07 | 0.01 |
|  | NorBC | 1.49 | 0.90 | 1.08 | 0.13 | 0.23 | 0.12 | 0.08 | 0.18 | 0.05 |
|  | NosZ | 0.07 | 0.04 | 0.09 | 0.08 | 0.28 | 0.05 | 0.10 | 0.24 | 0.03 |
|  | GS+=GOCAT | 0.64 | 0.54 | 0.49 | 0.28 | 0.45 | 0.26 | 0.28 | 0.46 | 0.13 |
|  | Ammonium transporter | 0.28 | 0.25 | 0.25 | 0.06 | 0.11 | 0.05 | 0.07 | 0.16 | 0.03 |
|  | **Total** | **6.01** | **3.80** | **4.65** | **0.93** | **1.71** | **0.72** | **0.96** | **1.65** | **0.45** |
| CBB | RuBisCO | – | – | – | 0.05 | 0.09 | 0.05 | 0.14 | 0.22 | 0.02 |
|  | Phosphoribulokinase | – | – | – | 0.11 | 0.08 | 0.10 | 0.09 | 0.11 | 0.10 |
|  | **Total** | **–** | **–** | **–** | **0.16** | **0.17** | **0.15** | **0.23** | **0.33** | **0.12** |
| rTCA | 2-oxoglutarate synthase | 0.57 | 0.45 | 0.47 | – | – | – | – | – | – |
|  | ATP citrate lyase | 0.64 | 0.59 | 0.52 | – | – | – | – | – | – |
|  | Pyruvate synthase | 0.47 | 0.40 | 0.47 | – | – | – | – | – | – |
|  | **Total** | **1.68** | **1.44** | **1.46** | **–** | **–** | **–** | **–** | **–** | **–** |
