# Supplementary material for: Physiological dynamics of chemosynthetic symbionts in hydrothermal vent snails
Source: ISME J. 2020 Jul 2;14(10):2568–79. doi: 10.1038/s41396-020-0707-2 (PMC7490688; doi:10.1038/s41396-020-0707-2)
Supplement: Supplementary file 5 — Supplementary Figure Legends [file 41396_2020_707_MOESM5_ESM.docx]

**Supplementary Figure Legends**

**Fig. S1** Schematic overview of the high-pressure respirometry experiments.

**Fig. S2** Concentrations of (a) H_2_S and (b) O_2_ during the separately conducted empty control experiment. The input water and three separate empty control aquaria were sampled for H_2_S concentrations during this experiment, but O_2_ was only able to be measured in the input water and in only one empty control aquarium.

**Fig. S3** Concentration of O_2_ in the input and effluent water during the treatment period in each experiment.

**Fig. S4** Concentrations of H_2_ and H_2_S in the input and effluent water during the treatment period in each experiment. Since empty controls could not be included in the H_2_S experiments, control values were estimated in a separate H_2_S treatment that replicated the original conditions.

**Fig. S5** Atom percent (A%) ^13^C values for the gill tissue of (a) *A. boucheti*, (b) *A. kojimai* and (c) *A. strummeri* in both the acclimation and treatment periods for each experiment. Black horizontal lines are the mean A% for the acclimation individuals and error bars are given as two standard deviations.

**Fig. S6** Transcriptome profiles for the (a) *A. kojimai*, (b) *A. strummeri* and (c) *A. boucheti* symbiont. Plotted values are TMM normalized TPMs.

**Fig. S7** Differential gene expression in the sulfide treated symbionts of (a) *A. kojimai*, (b) *A. strummeri* and (c) *A. boucheti*.

**Fig. S8** Differential gene expression in the hydrogen treated symbionts of (a) *A. kojimai*, (b) *A. strummeri* and (c) *A. boucheti*.
