# Supplementary figures and images for: Physiological dynamics of chemosynthetic symbionts in hydrothermal vent snails
Source: ISME J. 2020 Jul 2;14(10):2568–79. doi: 10.1038/s41396-020-0707-2 (PMC7490688; doi:10.1038/s41396-020-0707-2)

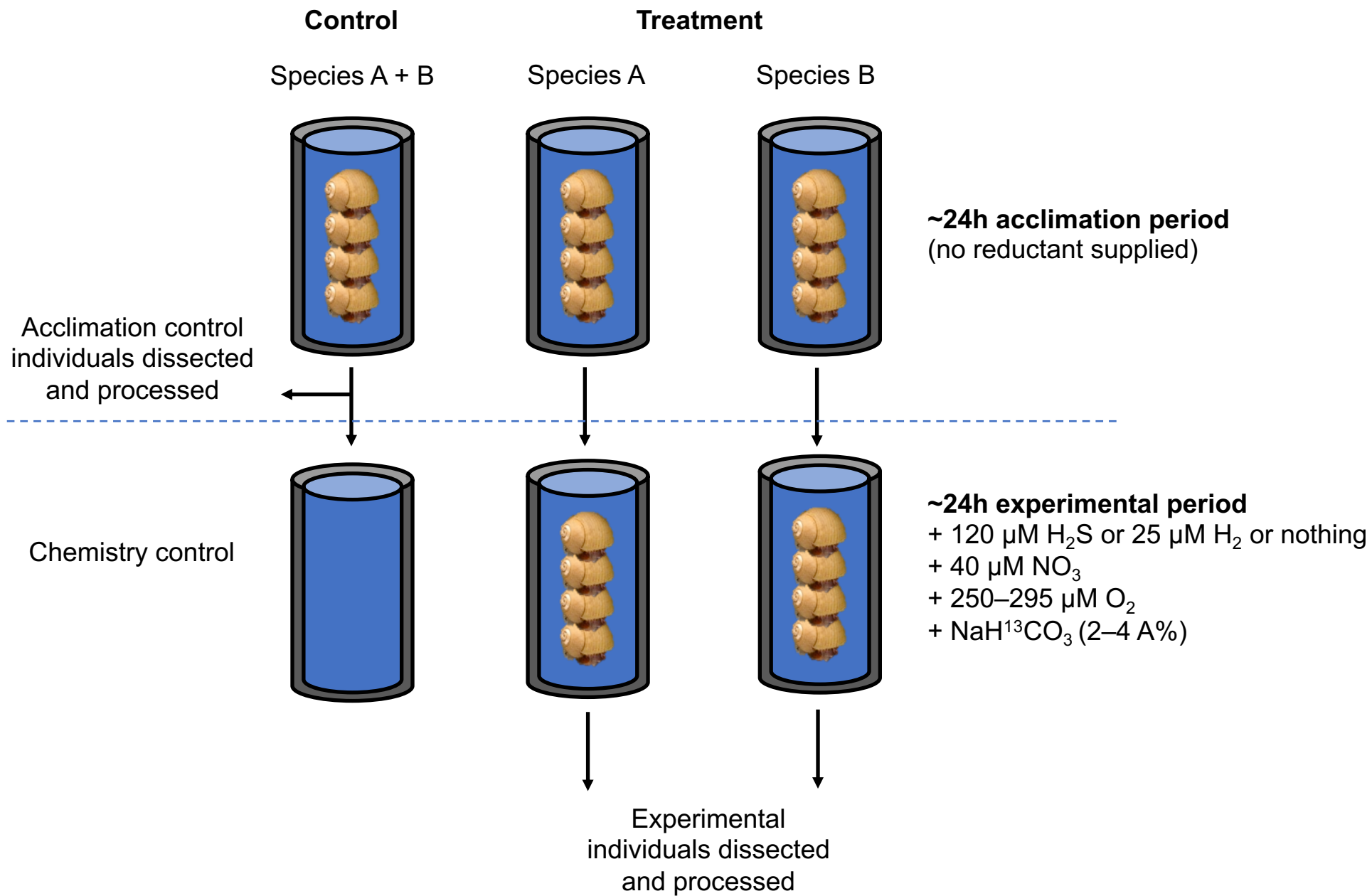

Supplement: Supplementary file 6 — Figure S1 [file 41396_2020_707_MOESM6_ESM.pdf]

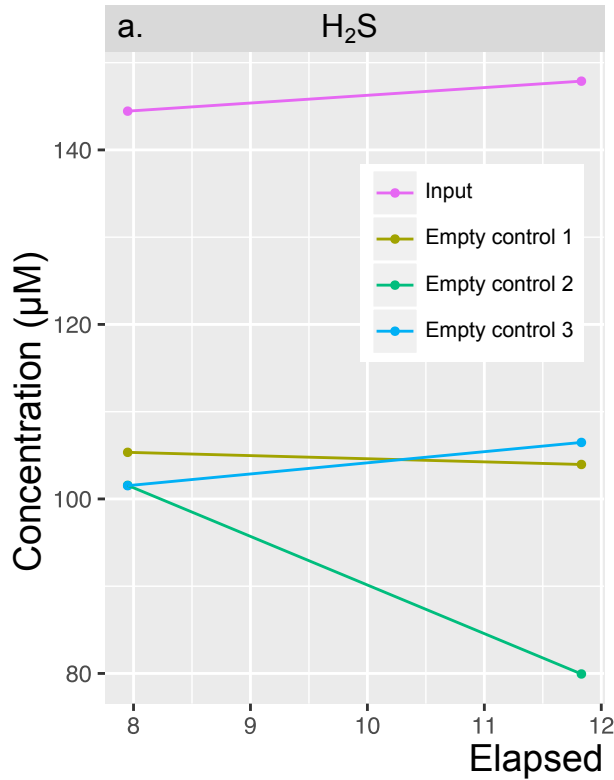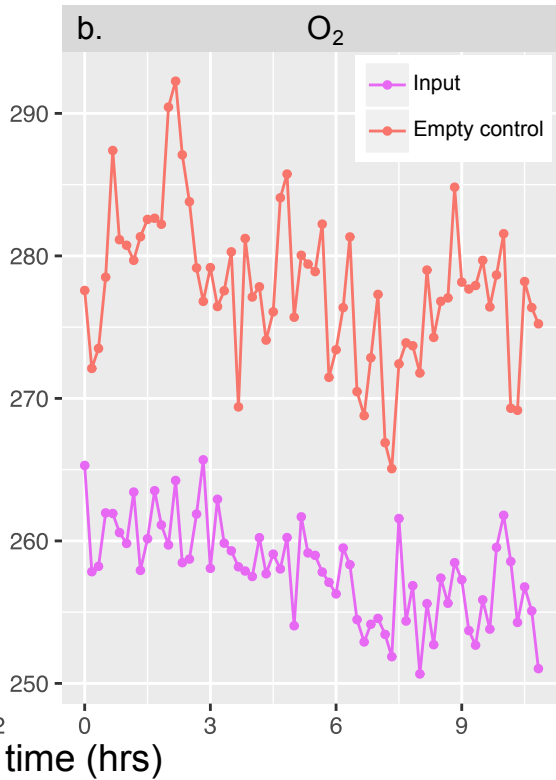

Supplement: Supplementary file 7 — Figure S2 [file 41396_2020_707_MOESM7_ESM.pdf]

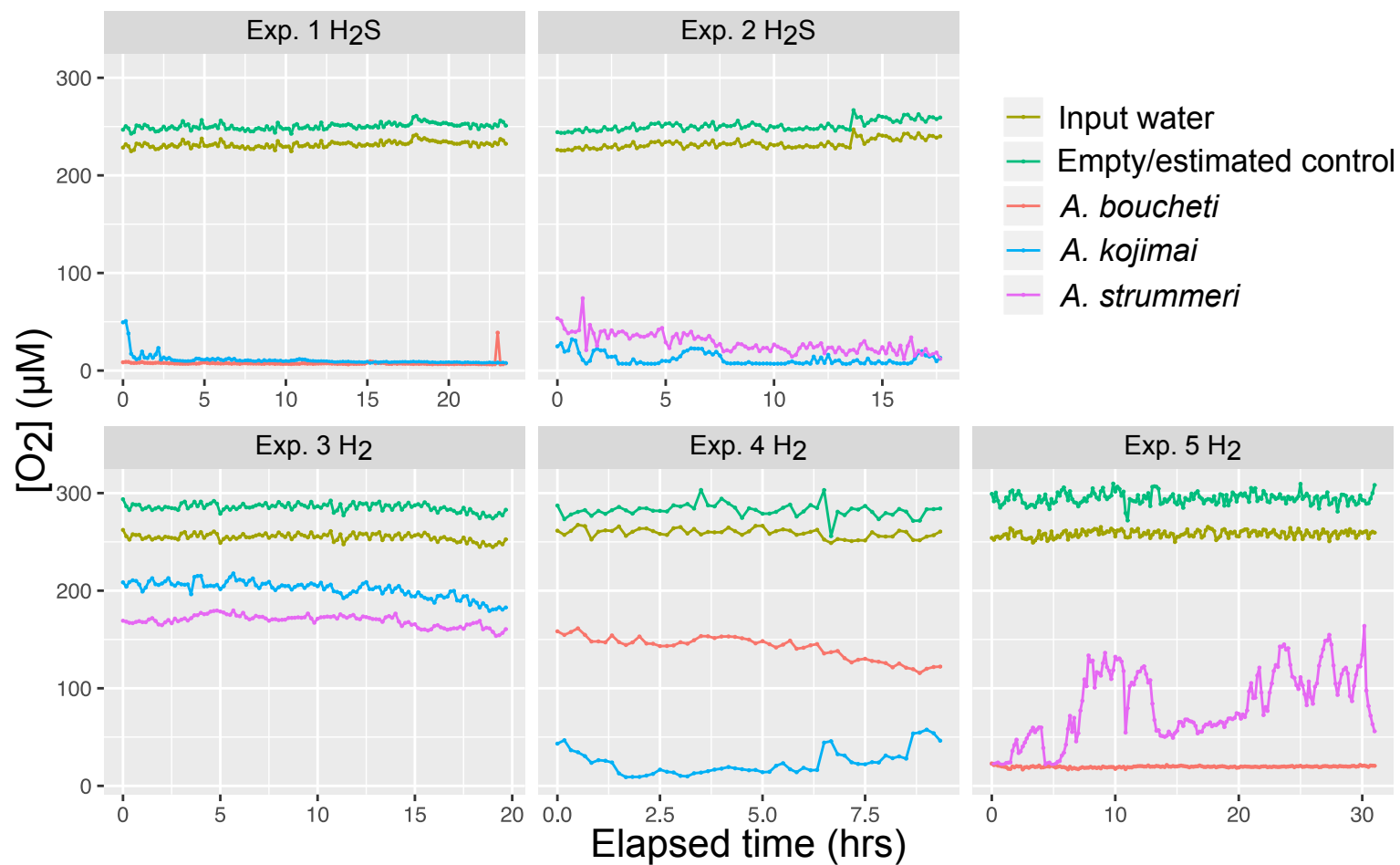

Supplement: Supplementary file 8 — Figure S3 [file 41396_2020_707_MOESM8_ESM.pdf]

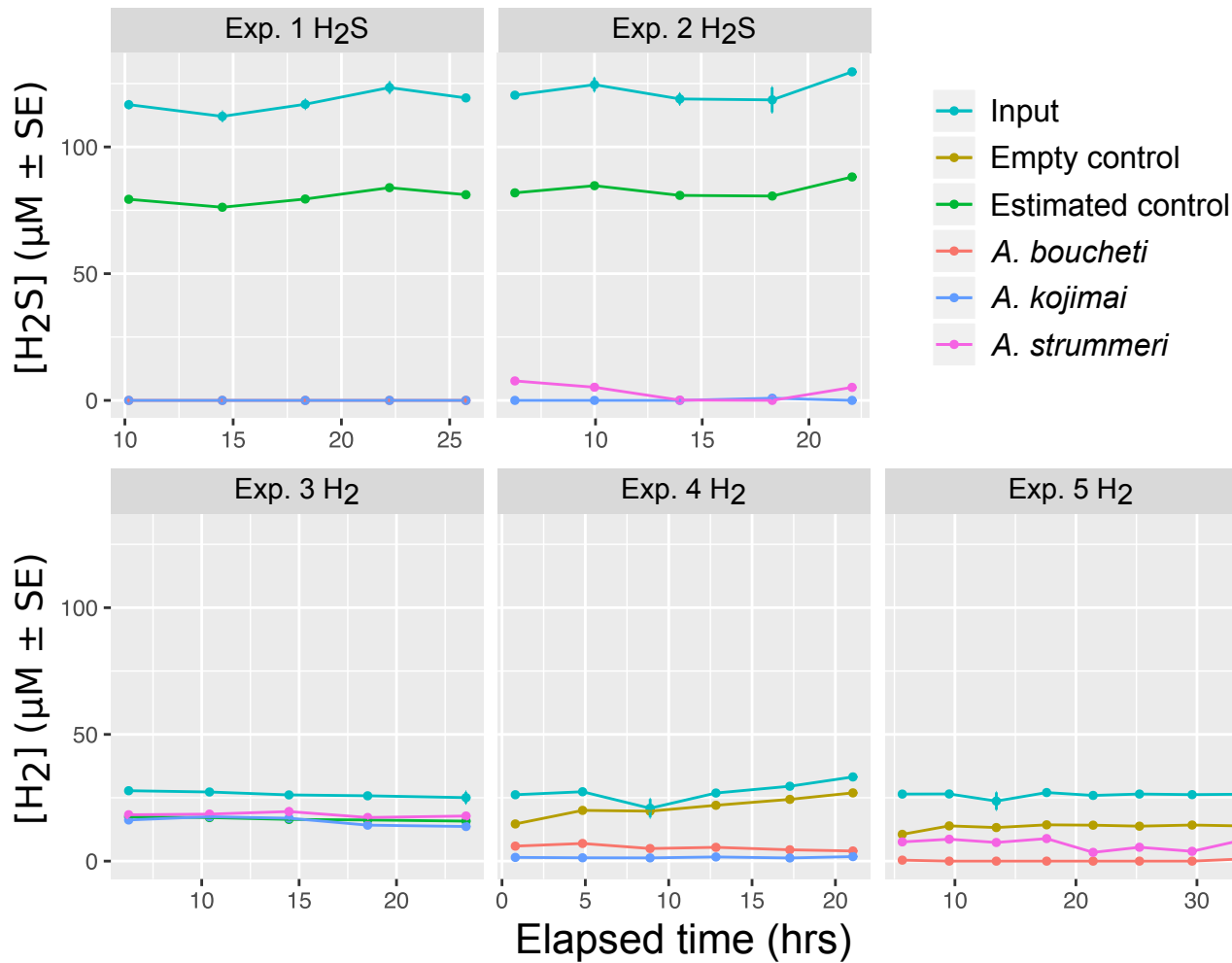

Supplement: Supplementary file 9 — Figure S4 [file 41396_2020_707_MOESM9_ESM.pdf]

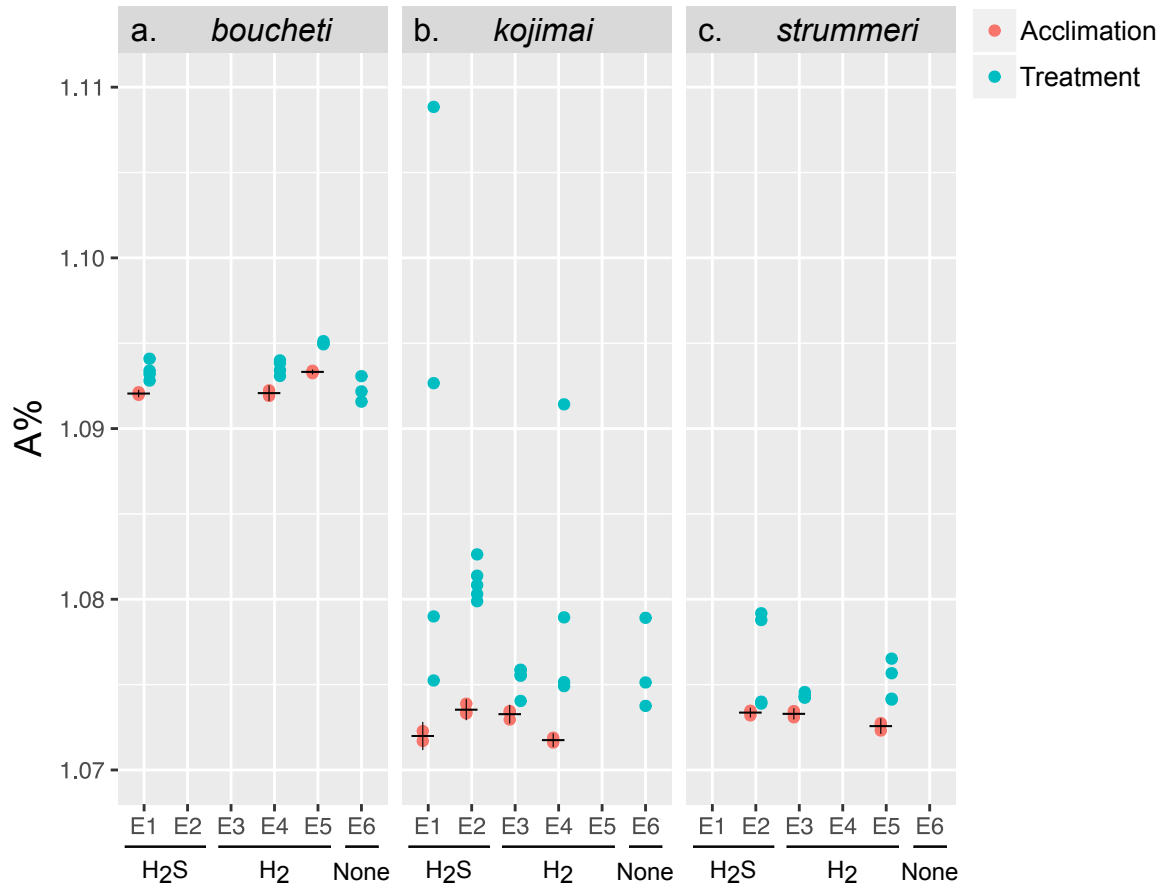

Supplement: Supplementary file 10 — Figure S5 [file 41396_2020_707_MOESM10_ESM.pdf]

**A** *A. kojimai***B** *A. strummeri***C** *A. boucheti*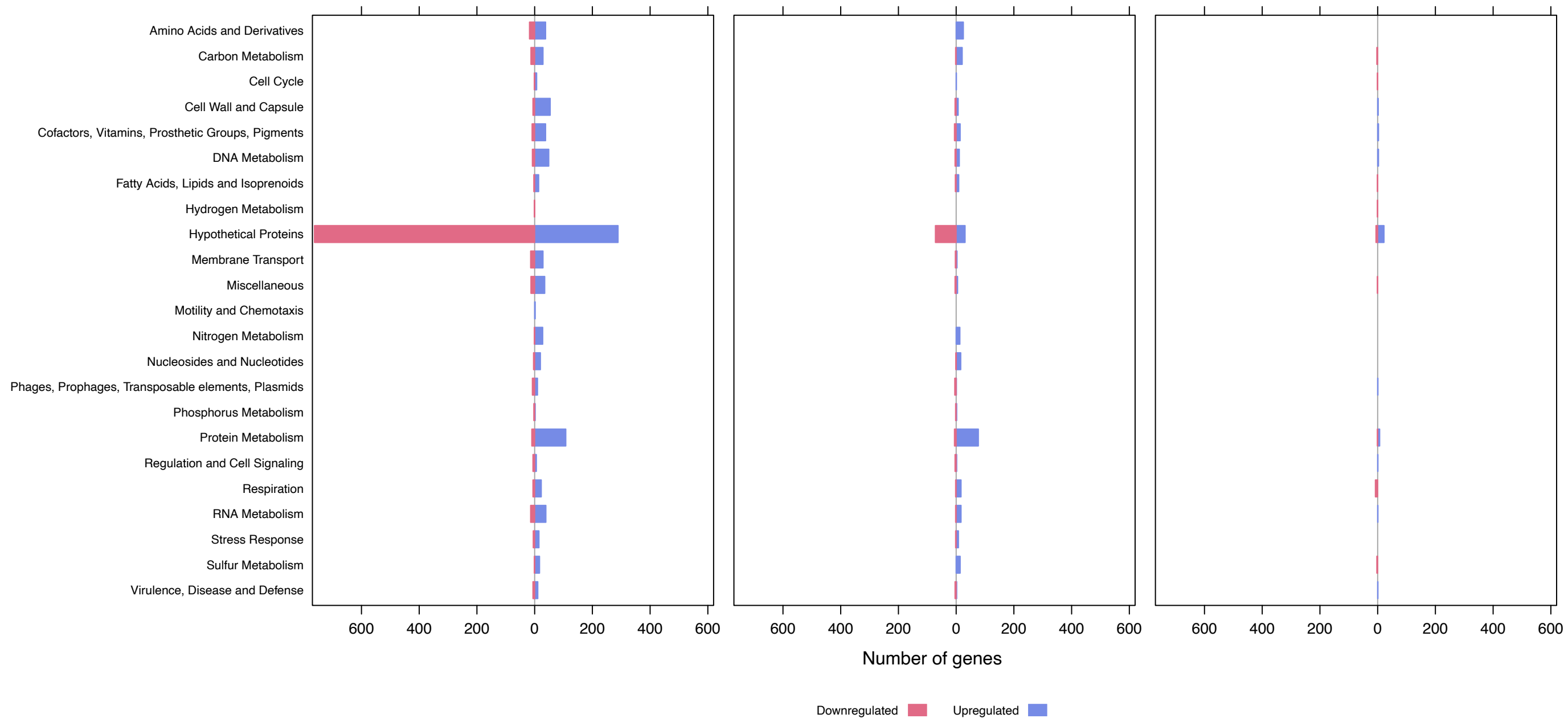

Supplement: Supplementary file 12 — Figure S7 [file 41396_2020_707_MOESM12_ESM.pdf]

**A** *A. kojimai***B** *A. strummeri***C** *A. boucheti*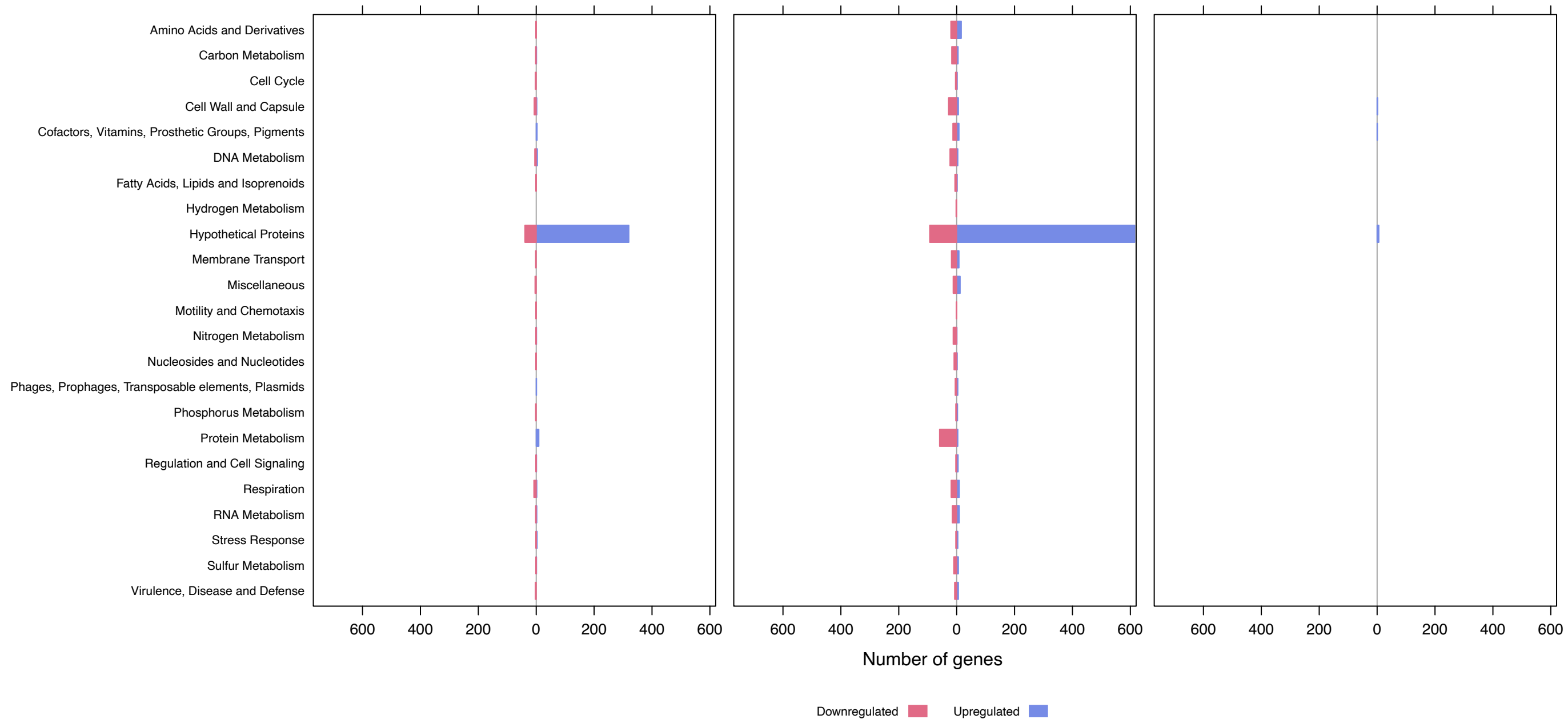

Supplement: Supplementary file 13 — Figure S8 [file 41396_2020_707_MOESM13_ESM.pdf]
