# Supplementary material for: Physiological dynamics of chemosynthetic symbionts in hydrothermal vent snails
Source: ISME J. 2020 Jul 2;14(10):2568–79. doi: 10.1038/s41396-020-0707-2 (PMC7490688; doi:10.1038/s41396-020-0707-2)
Supplement: Supplementary file 11 — Figure S6 [file 41396_2020_707_MOESM11_ESM.pdf]

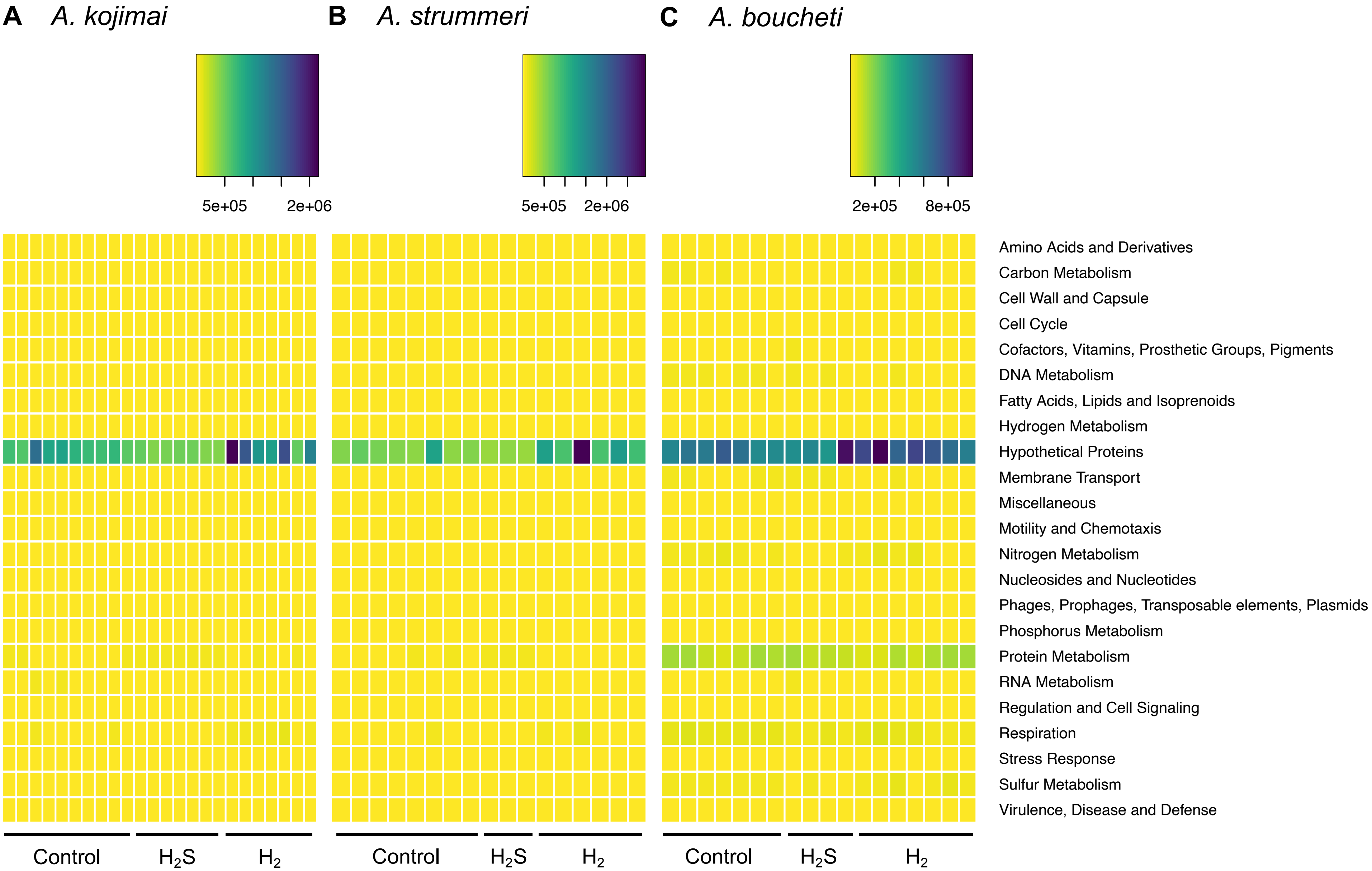
A A. kojimai
B A. strummeri
C A. boucheti
5e+05
2e+06
5e+05
2e+06
2e+05
8e+05
Amino Acids and Derivatives
Carbon Metabolism
Cell Wall and Capsule
Cell Cycle
Cofactors, Vitamins, Prosthetic Groups, Pigments
DNA Metabolism
Fatty Acids, Lipids and Isoprenoids
Hydrogen Metabolism
Hypothetical Proteins
Membrane Transport
Miscellaneous
Motility and Chemotaxis
Nitrogen Metabolism
Nucleosides and Nucleotides
Phages, Prophages, Transposable elements, Plasmids
Phosphorus Metabolism
Protein Metabolism
RNA Metabolism
Regulation and Cell Signaling
Respiration
Stress Response
Sulfur Metabolism
Virulence, Disease and Defense
Control
H_2S
H_2
Control
H_2S
H_2
Control
H_2S
H_2
